# Decoding High‐voltage LiCoO_2_: From Degradation to Stabilization Toward Durable Li‐ion Batteries

**DOI:** 10.1002/adma.202523570

**Published:** 2026-02-18

**Authors:** Zezhou Lin, Yiran Ying, Huangxu Li, Yanhao Ren, Tiancheng Liu, Peiyu Hou, Haitao Huang

**Affiliations:** ^1^ Department of Applied Physics The Hong Kong Polytechnic University Hong Kong China; ^2^ Research Institute for Smart Energy The Hong Kong Polytechnic University Hong Kong China; ^3^ School of Physics and Technology University of Jinan Jinan Shandong China

**Keywords:** electrode manufacturing, high‐voltage cathode, industrialized challenge, lithium ion batteries, modification strategy

## Abstract

Li‐ion batteries (LIBs) employing the commercially established LiCoO_2_ (LCO) cathode continue to dominate the market for portable electronic devices. Enhancing their volumetric energy density is crucial for extending the operational duration of advanced smart devices. One direct approach to increasing both specific capacity and energy density involves elevating the cut‐off charging voltage to above 4.6 V (vs Li/Li^+^). However, high‐voltage operation induces severe material degradation and battery failure, impeding further development of high‐voltage LCO technologies. This review first emphasizes the growing necessity for high‐voltage cathodes in contemporary LIBs, followed by a detailed exploration of the failure mechanisms of LCO at voltages up to 4.6 V. A systematic evaluation of emerging stabilization strategies is provided, covering foreign‐ion (co‐)doping, surface modifications, structural design, and electrolyte additives, all aimed at enhancing their structural integrity and electrochemical performance. Innovative battery design approaches and modification strategies for LCO‐based full cells are also discussed. Finally, the review concludes by identifying key scientific challenges and proposing targeted research avenues to enable high‐energy and durable LIBs using high‐voltage LCO. This review aims to offer guiding principles with significant implications for the rational design and development of high‐voltage cathode materials for advanced LIBs.

## Graphical abstract

This review systematically addresses the degradation mechanisms and stabilization strategies for high‐voltage LiCoO_2_ cathodes. Key enhancement approaches including foreign‐ion doping, surface modifications, structural design, and electrolyte optimization are critically assessed. Emphasis is placed on practical full‐cell integration and scalable manufacturing, bridging material advances with commercial high‐energy‐density battery development. Remaining challenges and future perspectives of high‐energy‐density battery are also outlined.

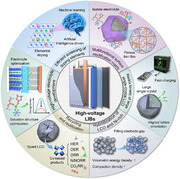

## 1 Introduction

The stability of Earth's environment faces serious challenges due to escalating energy waste and the intensifying greenhouse effect, which collectively pose risks to human living conditions [1, 2, 3]. In response to these environmental issues, worldwide efforts are increasingly directed toward sustainable energy utilization practices. The creation of sustainable energy frameworks requires urgent research attention, with special emphasis on enhancing energy harvesting, conversion, and storage methods to reach both economic sustainability and operational effectiveness [4, 5, 6]. Additionally, developing clean and high‐performance energy storage options is crucial for supporting the ongoing needs of transportation, digital infrastructure, and urban energy management in today's interconnected communities. Although portable electronics have experienced rapid expansion and continuous upgrading in both hardware and software components throughout the last ten years, advancements in portable energy storage technologies have progressed at a more measured pace [7, 8, 9, 10].

**FIGURE 1:**
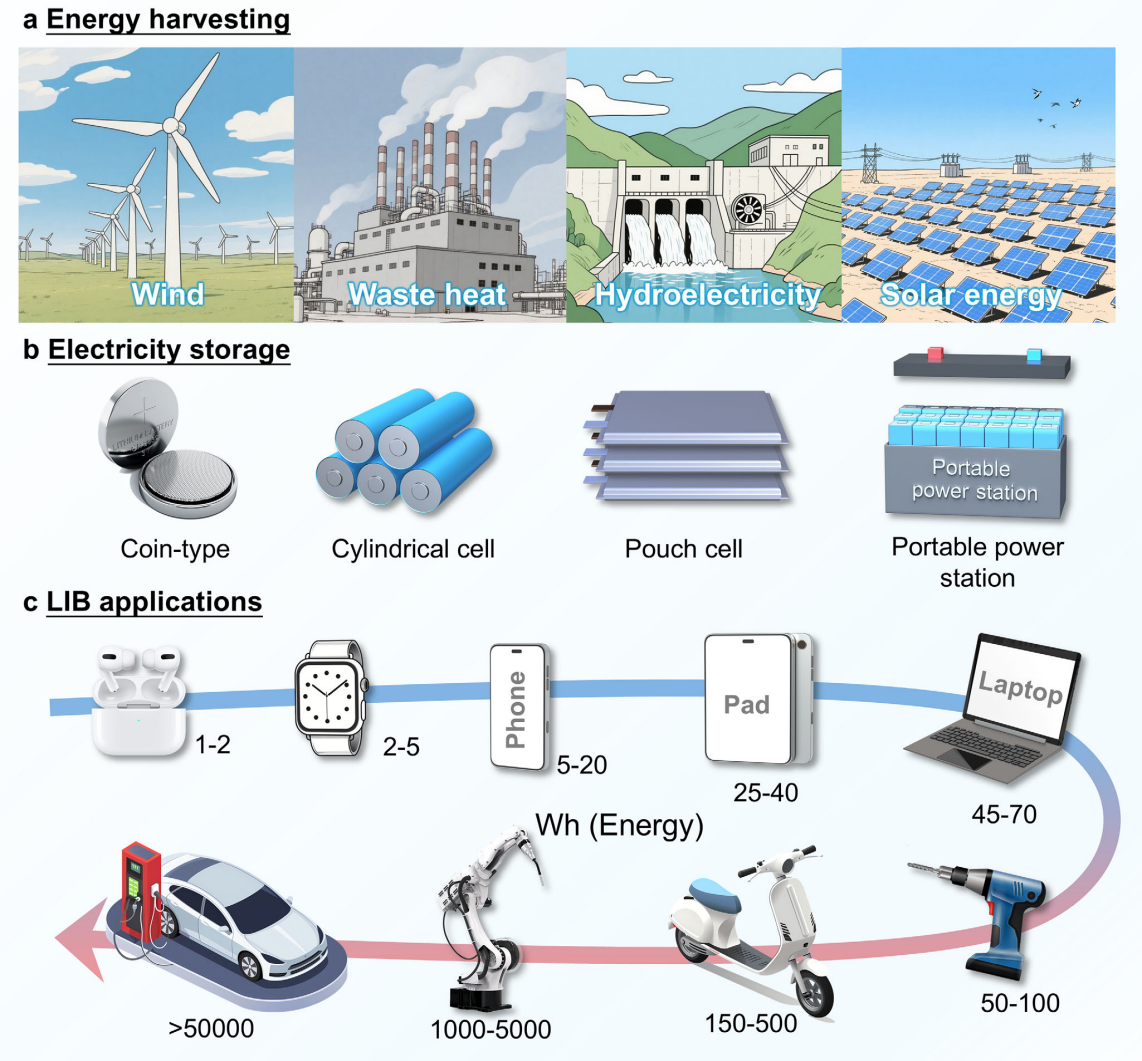
The modern sustainable energy system, including (a) energy harvesting, (b) electricity storage, and (c) LIB applications in modern society.

The Industrial Revolution served as a crucial catalyst for societal progress while establishing more effective energy consumption and storage infrastructure. The transition toward environmentally friendly, sustainable energy sources alongside sophisticated energy storage systems has emerged as an unavoidable direction for contemporary societal evolution. As shown in Figure 1, modern sustainable energy system mainly consists of three components: (1) Renewable energy harvesting and conversion (wind, solar, hydroelectric, and thermal sources), where captured energy is primarily stored as electrochemical energy. (2) Advanced energy storage technologies, particularly rechargeable batteries that enable highly efficient bidirectional conversion between electrical and chemical energy through reversible transition metal redox reactions. (3) Efficient energy applications, where the proliferation of portable electronics and electrified transportation has driven unprecedented demand for compact, high‐performance battery systems. Currently, rechargeable batteries find broad applications across multiple fields including mobile phones, portable computers, electrical equipment, and electric transportation systems [11, 12, 13, 14].

Within the landscape of electrical energy storage technologies, lithium‐ion batteries (LIBs) have consistently emerged as the preferred solution for portable electronics, owing to their compact form factor and superior energy density [14, 15]. Entering the 21^st^ century, LIB technology has progressed at a remarkable pace, marked by continual miniaturization, rising energy densities, and improved operational reliability. These attributes closely match the demands of more lightweight and compact smart devices [16, 17, 18]. A key contributor to these improvements has been the evolution of layered cathode materials, which demonstrate outstanding reversibility during cycling. Lithium (Li), being the lightest metal, fundamentally enable the exceptional energy density that distinguishes modern LIBs. Following the commercial debut in 1991, LIBs have undergone transformative technological evolution, becoming indispensable advanced energy storage devices. The operating principle of LIBs involves the reversible extraction and insertion of Li^+^ ions from and into the cathode host structure, enabling the interconversion of electrical and chemical energy. Battery energy density is mathematically defined as the product of reversible capacity and operating voltage. Therefore, increasing the charge cut‐off voltage directly leads to a substantial improvement in overall energy density [19, 20, 21]. For layered transition metal oxides [22], elevating the charging cutoff potential from 4.2 V (50% Li^+^ extraction) to 4.6 V (80% Li^+^ extraction) allows for more Li^+^ extraction/insertion, thereby achieving energy densities exceeding 700 Wh L^−1^. This voltage engineering approach represents a critical pathway for advancing high‐energy‐density storage systems compatible with renewable energy integration.

Looking beyond portable electronics, LIBs are positioned to serve as sustainable energy storage devices in a wider range of future applications. Their broader adoption can support the transition toward sustainable and intelligent societies. Realizing such sustainable energy goals requires in‐depth study of battery operating mechanisms, degradation pathways, and innovative cell designs to create optimized storage systems. Through continued fundamental research, it becomes possible to develop eco‐friendly and high‐efficiency cathode materials that deliver improved capacity and cycle life, while also reducing production costs and streamlining manufacturing procedures. Furthermore, as human activities extend into more challenging environments, LIBs must operate safely and reliably under extreme conditions, such as wide temperature ranges, intense electromagnetic fields, and high external stress [23, 24]. These harsh environments can trigger catastrophic failures, including thermal runaway, internal short circuits, and hazardous gas generation, which also lie at the root of numerous safety incidents in consumer electronics [25, 26]. Therefore, future battery manufacturing should incorporate intrinsic engineering and systematic validation of electrodes, electrolytes, and their interfaces. This approach will ensure stable performance across diverse extreme environments, thereby overcoming a major obstacle to broader commercial adoption.

## 2 Requirement of High‐Voltage Cathodes

**FIGURE 2:**
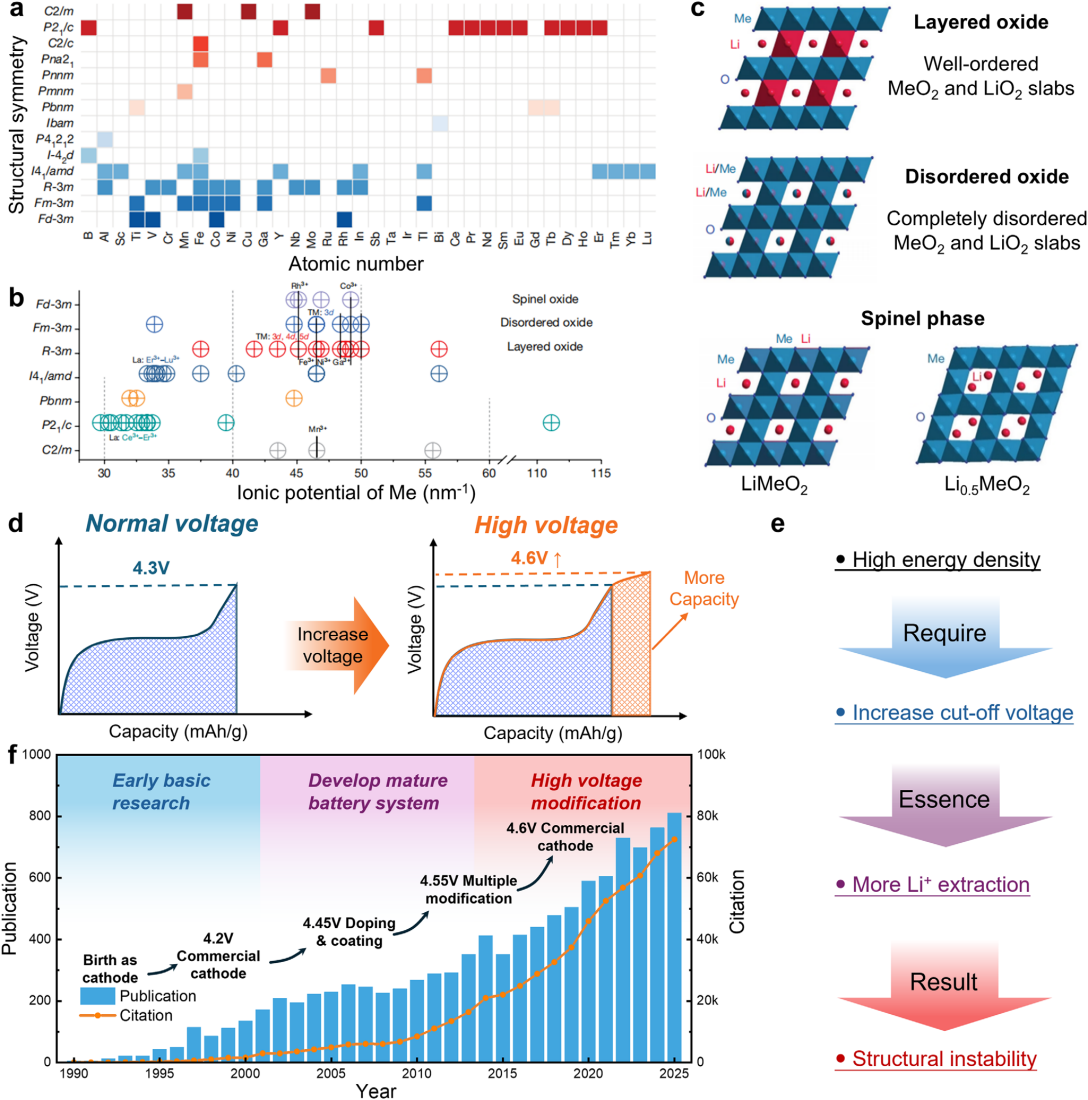
(a) Structural symmetry of LiTMO_2_ compositions. (b) Ionic potential of TM ions. (c) Structure transformation in layered, disordered and spinel oxides. Reproduced with permission [39]. Copyright 2024, Springer Nature. (d) Higher voltage brings more capacity. (e) High‐voltage results in structural instability. (f) Increasing citations and publications of LCO study from 1990 to 2025. The data are from Web of Science using the search topic of “LiCoO_2_”.

The widespread adoption of rechargeable battery technology serves as a fundamental enabler for modern societal progress. Following Sony's successful commercialization of LIBs in 1991, LCO has emerged as the predominant commercial cathode material [14]. To enhance the energy density and lower manufacturing costs of LIBs, significant efforts have been directed toward the creation of novel cathode materials [27, 28, 29, 30]. Scientists have developed numerous cathode material families, including LiFePO_4_ (LFP) [31, 32], LiMn_2_O_4_ (LMO) [33], LiNiO_2_ [34], LiNi_1−_ *_x_*_−_ *_y_* Co*_x_* Mn*_y_* O_2_ (NCM) [35, 36], LiNi_0.5_Mn_1.5_O_4_ [37] and LiNi_0.80_Co_0.15_Al_0.05_O_2_ (NCA) [38]. These diverse cathode materials have been deployed across various sectors of contemporary society according to their specific performance characteristics. Particularly, LMO, LFP, NCM, and NCA have seen substantial implementation in electric vehicles and intelligent energy storage installations. As shown in Figure 2, LiTMO_2_ compound family constitutes an extensive materials system where strategic substitution of transition metal (TM) ions enables tuning of crystal structures and electrochemical charge/discharge characteristics [39]. Within this spectrum of cathode options, LCO maintains distinct advantages including high Li^+^ ion and electronic conductivity, elevated density (4.2 g cm^−3^), outstanding cycling durability, and proven safety characteristics. These exceptional properties collectively explain why LCO continues to serve as the principal cathode material for portable electronic devices [40].

LIBs have become deeply integrated into daily life, driving substantial research focus on extending battery service life and enhancing usable capacity [41]. Figure 2f demonstrates that scientific investigation of LCO cathode materials has grown exponentially since its inception, progressing from fundamental studies to mature battery technology and recently to high‐voltage structural modifications. Developing lightweight LIBs necessitates substantial improvement in the reversible capacity of LCO cathodes. Conventional LCO systems operating at 4.2 V (vs Li/Li^+^) deliver approximately ∼140 mAh g^−1^, representing only 51% of the theoretical capacity of 274 mAh g^−1^. This substantially unexplored capacity indicates that nearly half of the Li^+^ ions remain inactive within the Li*_x_* CoO_2_ framework. As a technology compatible with existing manufacturing infrastructure, raising the upper voltage threshold stands as the most practical approach to capacity improvement, though this requires the cathode to endure more profound Li^+^ extraction and insertion processes. As charging voltages progressively increase over time, LCO demonstrates considerable potential for energy density enhancement. Specifically, LCO delivers energy densities of 740 Wh kg^−1^ at 4.45 V and 840 Wh kg^−1^ at 4.55 V [42]. Combined with the exceptional tap/compaction density of single‐crystal LCO, these characteristics enable LIBs with superior volumetric energy density, which is a decisive advantage for portable power applications.

The rapid growth of electric vehicles and miniaturized Computer‐Communication‐Consumer (3C) electronics (i.e., laptops, smartphones, and wearables) has created unprecedented demand for advanced energy storage solutions. Particularly with the advent of the 5G era, mobile phones require faster processing and transmission speeds, posing challenges for energy storage batteries in terms of small capacity, short battery life, and slow charging. To address these issues, there is a need to develop new high‐performance LIBs with high volumetric energy density, fast‐charging capability, and long cycle lifespan [43]. In the domain of 3C applications specifically, current research emphasizes the optimization of cathode materials to improve both volumetric capacity and high‐rate performance without compromising operational safety. These advancements have become increasingly urgent in light of the exponentially rising power demands of contemporary portable electronic devices [44].

## 3 Failure Mechanisms of LCO and LIBs Under High‐Voltage Operation

**FIGURE 3:**
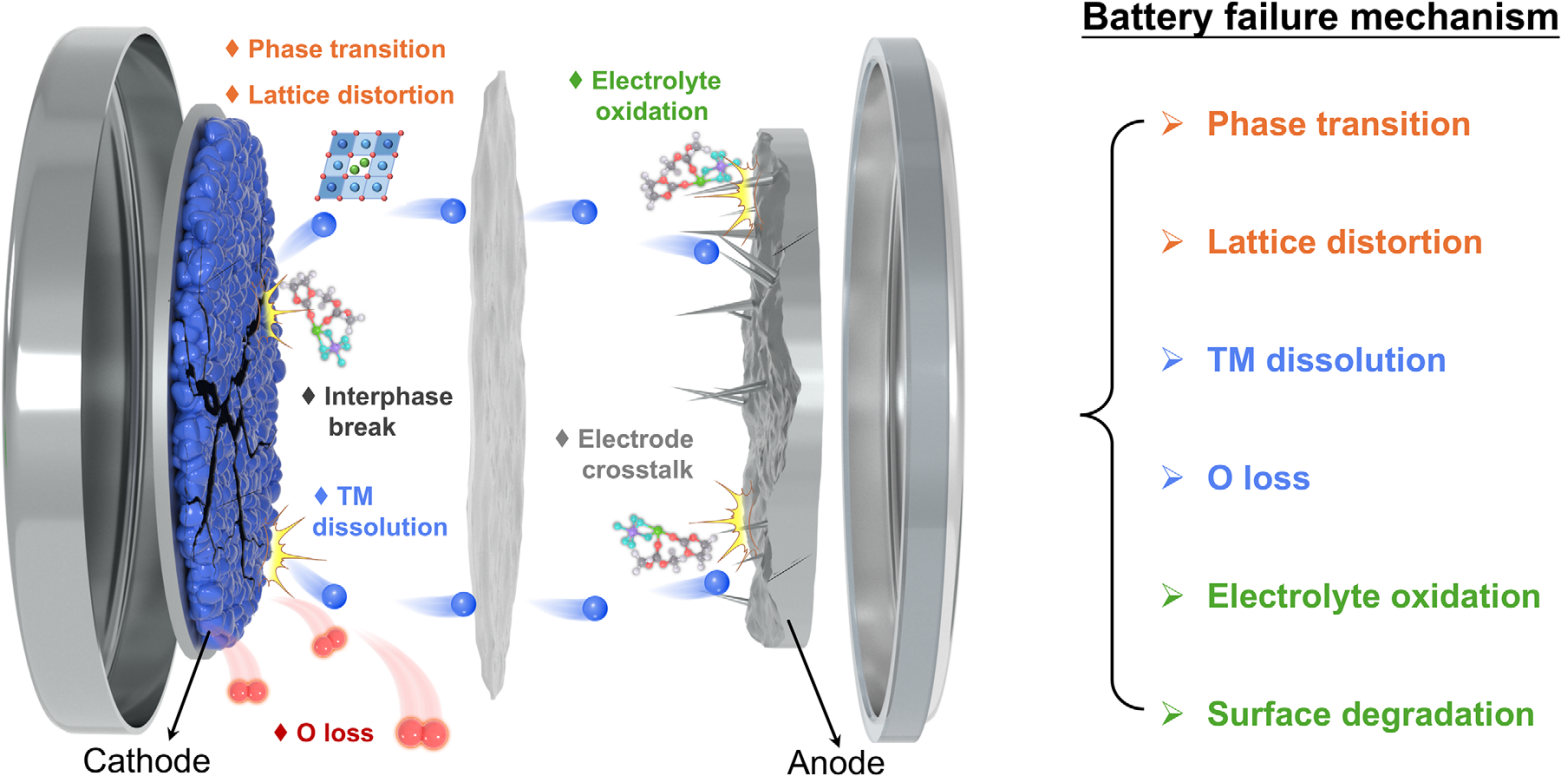
Schematic diagram of the failure process of LIBs as LCO cathode under high‐voltage.

While operating LIBs at elevated voltages allows access to higher reversible capacity and improved energy density, this approach also accelerates performance decay. Recent studies focusing on degradation pathways in high‐voltage LCO systems have identified several critical failure mechanisms that must be resolved to advance next‐generation battery technology [45, 46]. As summarized in Figure 3, the primary failure modes affecting LCO cathodes under high‐voltage conditions can be categorized into three interrelated processes: (1) irreversible phase transitions and lattice distortion, (2) TM ions dissolution and lattice oxygen loss, and (3) oxidative decomposition of the electrolyte and associated surface deterioration.

The first category involves structural changes including phase transitions and lattice distortion. As Li^+^ ions are extracted during charging, LCO experiences successive phase transformations that induce substantial volume variations within the material. These repeated volume changes generate mechanical stress, ultimately leading to particle fracture and accelerated capacity fade [47]. The second group of failure mechanisms relates to chemical instability, specifically dissolution of transition metal ions and oxygen loss from the lattice. High‐voltage operation destabilizes the surface structure of LCO, promoting dissolution of Co^2+^ ions into the electrolyte. Concurrently, surface oxygen species become increasingly unstable under high potentials, leading to oxygen release and progressive degradation of the host structure [48]. The third major challenge involves interfacial instability and uncontrolled side reactions. Under high‐voltage conditions, the organic electrolyte is prone to oxidative decomposition on the LCO surface, leading to the formation of a cathode‐electrolyte interphase (CEI) layer. An ideal CEI should be thin, ionically conducting, and mechanically stable to protect the cathode. However, the CEI formed under high voltage is often unstable and thick. It tends to fracture during cycling due to the repeated volume changes of LCO. Each fracture exposes fresh cathode surface, triggering new CEI formation. This process continuously consumes Li^+^ ions and electrolyte. Consequently, the CEI layer keeps growing, leading to a significant increase in impedance and accelerated capacity fade [49]. A comprehensive understanding of these failure processes provides essential insights for designing more robust cathode materials and developing strategies to enhance overall battery performance.

### 3.1 Phase Transition and Lattice Distortion

**FIGURE 4:**
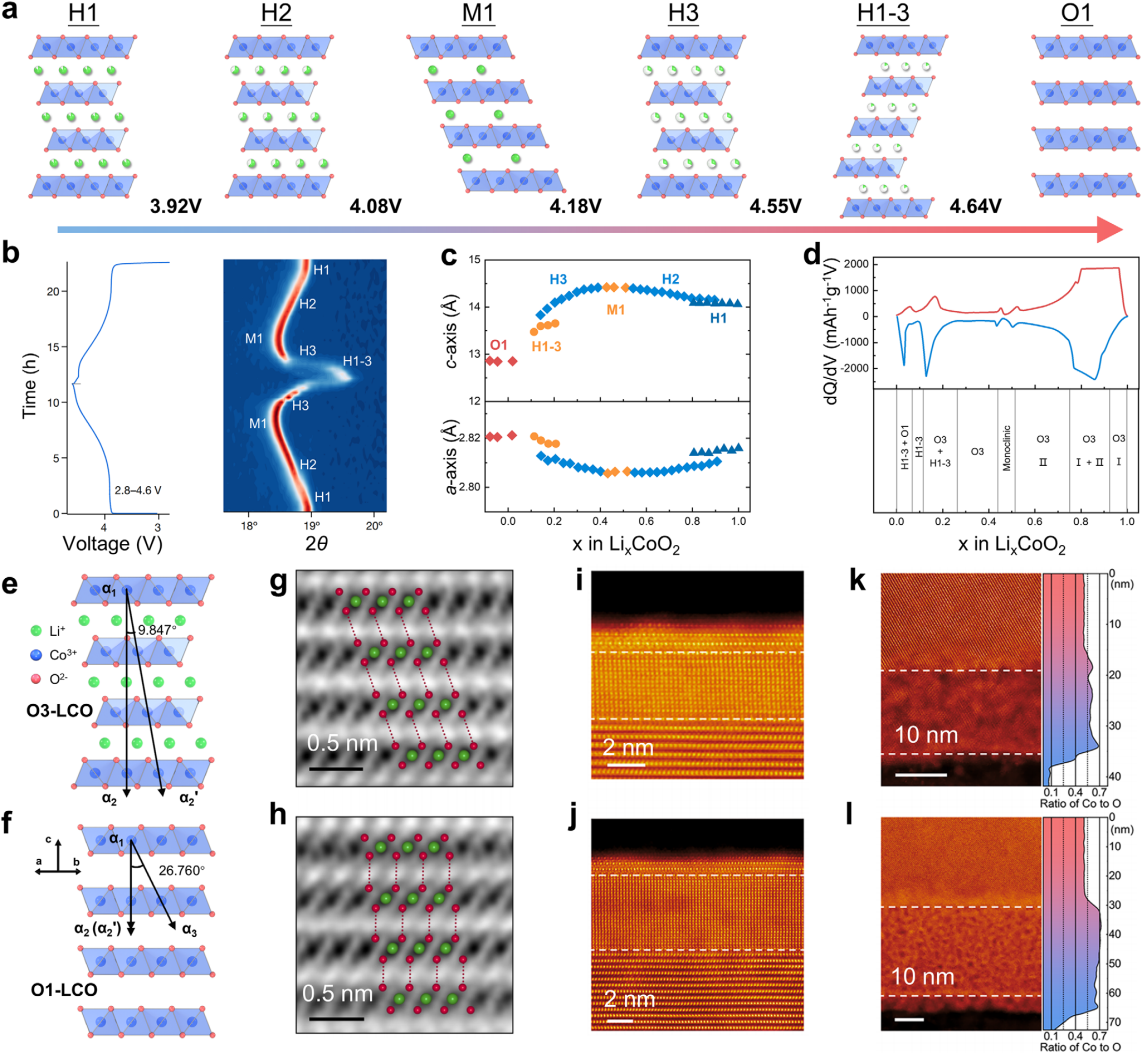
(a) The structure changes of LCO with varying degrees of Li^+^ extraction under different charging voltages. (b) In situ X‐ray diffraction (XRD) analysis of (003) peak alongside the corresponding charge‐discharge profile positioned to the left. Reproduced with permission [39]. Copyright 2024, Springer Nature. (c) Variation of *c*‐ and *a*‐axis dimensions. Reproduced with permission [22]. Copyright 1996, The Electrochemical Society. (d)  Corresponding structure. Reproduced with permission [54]. Copyright 2004, Elsevier. Structural illustration of (e) O3 and (f) O1 phase in LCO. Atomic resolution ADF‐STEM images showing the surface atomic structures of LCO charged to (g) 4.6 and (h) 4.8 V, respectively. Representative ADF‐STEM images and corresponding depth‐dependent Co/O ratio of LCO particles discharged from (i) 4.6 and (j) 4.8 V charged states, respectively. (k, l) Depth‐resolved electron energy loss spectroscopy (EELS) of the O *K*–edge and Co *L*–edge from the surface (bottom) to the bulk (top) with a step of 1 nm. Reproduced with permission [55]. Copyright 2025, American Chemical Society.

Two distinct electrochemical regions in structural evolution of Li*_x_* CoO_2_ are shown in Figure 4a [50]. During low‐voltage operation (0.45 ≤ *x* ≤ 1), delithiation induces an initial insulator to metal transition (*x* = 0.93→0.75) through electron delocalization [22]. Then, when charged to 4.2 V, approximately 50% of the Li^+^ ions are extracted and the material experiences an order–disorder transition (hexagonal–monoclinic–hexagonal phase) [51]. The emergence of the monoclinic phase is widely recognized as a source of structural deterioration and represents one key reason for capacity reduction when operating above 4.2 V [52, 53]. In the high‐voltage region (*x* < 0.45), excessive Li^+^ extraction drives an irreversible O3→H1‐3/O1 phase transition in LCO [54], where the metastable H1‐3 phase (mixture of O1 and O3) forms through interlayer O─Co─O slab gliding and Li vacancy ordering. This structural reorganization generates substantial anisotropic lattice stress, while simultaneously impeding Li^+^ mobility through disrupted diffusion channels. These phase transitions, combined with decreased Li^+^ diffusivity and increased mechanical stress, result in dramatic capacity fading in deeply charged Li*_x_* CoO_2_. Consequently, the charging voltage was restricted to 4.2 V during the early development stages of commercial LCO‐based batteries.

During high‐voltage cycling, the development of a stripe‐like O1 phase along LCO grain boundaries further initiates irreversible transformations in the bulk material. In addition, repeated charge‐discharge cycles induce large variations in the *a*‐axis and *c*‐axis lattice parameters, resulting in interlayer sliding, mechanical cracking, and microcrack formation within the electrode [47]. These structural changes, closely linked to phase transitions, play a central role in capacity fade and overall performance decay of LCO. A thorough understanding of these degradation mechanisms is essential for guiding the design of more reliable LIBs.

Phase transitions below 4.5 V exhibit reversibility with negligible capacity loss [56], whereas charging exceeding 4.6 V triggers irreversible structural degradation, combined with a low Li^+^ ion diffusion coefficient [57]. At deep delithiation (*x* < 0.4), the contracted interlayer spacing in H1‐3/O3 phases (Figures 4b–d) increases Li^+^ diffusion barriers, severely restricting ionic transport. Concurrently, accumulated mechanical stress from repeated phase transformations induces microstructural defects and surface reconstruction, creating electrochemically inactive domains that progressively reduce reversible capacity. Ultimately, such structural degradation negatively affects both the practical performance and long‐term cycling stability of LCO cathodes.

As shown in Figures 4e,f, experimental observations clearly demonstrate that the bulk crystal structure of LCO experiences significant modification when charged beyond 4.5 V. While substantial Li^+^ removal does not cause immediate capacity collapse, prolonged cycling under these conditions leads to cumulative volume changes and internal stress buildup. These effects eventually cause mechanical damage to the cathode structure. This degradation is especially apparent during the phase transitions occurring at 4.2 V, where a monoclinic phase forms, and at 4.55 V, where the H1‐3 phase emerges. Multiple investigations have verified that charging LCO beyond 4.55 V induces an O3 to H1‐3 phase transformation. This process involves considerable lattice contraction that markedly raises the risk of permanent damage to the bulk crystal framework. To quantitatively describe the underlying glide of CoO_6_ slabs, a gradual angle (δ) is defined as∠α_2_α_1_α_2’_ ∼ 9.847°, referring to the *‐αβγαβγ‐* stacking of Co ions in O3‐LCO along the [001] direction, while the ∠α_2_α_1_α_2’_ is equal to 0° in O1‐LCO with the *‐ααα‐* stacking of Co ions [58]. Such complex phase behavior considerably affects the electrochemical properties of the material. After charged to 4.8 V, the crystal structure cannot completely revert to its initial state, clarifying the atomic‐scale origin of capacity fading in LCO under high‐voltage cycling.

Interface deterioration also proceeds through surface phase transitions. As shown in Figures 4g–l, as evidenced by high‐angle annular dark field scanning transmission electron microscopy (ADF‐STEM), deep delithiation induces irreversible transformation of the surface layers from ordered layered to defective spinel configurations [55]. Notably, crystallographic anisotropy governs this process. At the 4.8 V charged state, the surface of the LCO material forms a distinct double‐layer structure, with the (003) crystal plane exhibiting a 2–3 nm thick double layer (including spinel and O1 phases), while the non‐(003) crystal plane shows only the spinel phase. Upon discharged to 3.0 V, the (003) crystal plane structure nearly fully recovers, whereas the non‐(003) crystal plane forms a thicker defective spinel phase (up to 5–8 nm), indicating that the different crystal planes exhibit markedly different structural reversibility. These results demonstrate that the surface structural degradation of LCO due to high‐voltage cycling is strongly dependent on the crystal plane, with the non‐(003) crystal plane being the primary area for irreversible structural changes. This selective degradation is related to the differences in electronic structure and chemical stability across different crystal planes. Therefore, suppressing the deleterious 4.55 V phase transition represents a critical design criterion for advanced cathode materials, as its effective mitigation directly enables stable high‐voltage LIB operation with significantly improved cycle life.

**FIGURE 5:**
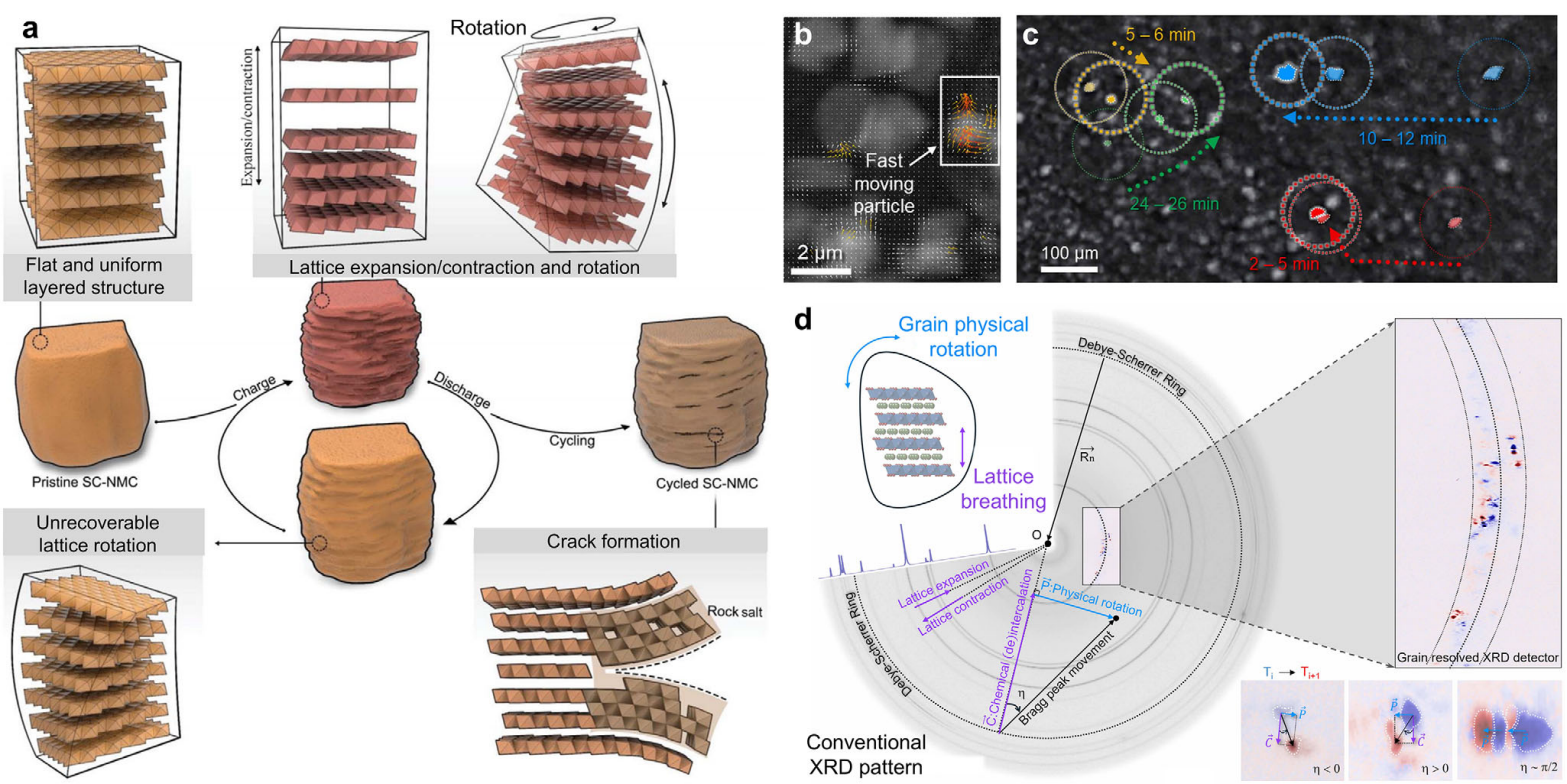
(a) Diagram depicting the structural degradation of cathodes. Reproduced with permission [59]. Copyright 2024, The American Association for the Advancement of Science. (b) Randomly occurred and sparsely distributed fast‐moving cathode particles. (c) Optical imaging of randomly occurred irregular particle movements. (d) Schematic illustration of the synchrotron‐based, grain‐resolved XRD. Reproduced with permission [60]. Copyright 2024, The American Association for the Advancement of Science.

Rotational lattice distortion has been identified within single‐crystal cathode particles as another critical mechanism responsible for electrode degradation [59]. As shown in Figure 5a, such lattice reorientation facilitates defect generation and provides a propagation pathway for structural imperfections during Li^+^ insertion and extraction. With continued cycling, the progressive buildup of these rotational distortions gradually degrades both the morphology and crystal integrity of single‐crystal particles, eventually causing irreversible mechanical failure. This lattice distortion initiates multiple damaging processes, including microcrack formation, non‐reversible phase changes, and surface reconstruction, which collectively contribute to substantial deterioration in electrochemical behavior. Since lattice distortion and strain inherently accompany electrochemical cycling in these materials, developing strategies to suppress these distortions becomes essential for enhancing the durability of single‐crystal cathodes and avoiding accelerated capacity loss.

Additionally, it has been demonstrated that cathode materials exhibit continuous strain dynamics during charge and discharge cycles. This phenomenon extends from the lattice expansion and contraction of individual particles to microscale stress and strain [60]. Figures 5b–d show the real‐time process of electrode particles moving closer together, rotating, and rearranging during the initial charging phase. Such localized deformation progressively propagates to surrounding regions, triggering a self‐organized reconstruction of the entire electrode. Furthermore, conventional electrode manufacturing methods (ball milling, calendering, and rolling, etc.) introduce additional crystallographic defects like slip bands, dislocations, lattice strain, kink boundaries, and microcracks [61, 62]. These processing‐induced defects act as initiation sites that expand during battery operation, ultimately accelerating both capacity fade and mechanical breakdown.

### 3.2 TM Dissolution and Oxygen Loss

During repeated charge and discharge cycles, LCO electrodes experience continuous Li^+^ ion insertion and removal, accompanied by cyclic structural phase changes. The surface region of cathode particles displays reduced structural stability compared to the bulk material, primarily due to the abundance of unsaturated chemical bonds. This inherent instability becomes especially pronounced under high‐voltage conditions, promoting dissolution of transition metal ions and release of lattice oxygen, which are two major drivers of surface breakdown [63]. Such structural and chemical transformations at the cathode surface critically impact the cycling stability of LCO. Specifically, the loss of Co and oxygen can trigger compositional changes, ultimately deteriorating its electrochemical performance and structural integrity [64]. These surface evolution processes directly contribute to capacity fading, mechanical damage, interface failure, and overall performance decline. Consequently, elucidating the fundamental mechanisms behind surface chemical and structural changes remains crucial for understanding the long‐term failure modes of LCO‐based battery systems.

**FIGURE 6:**
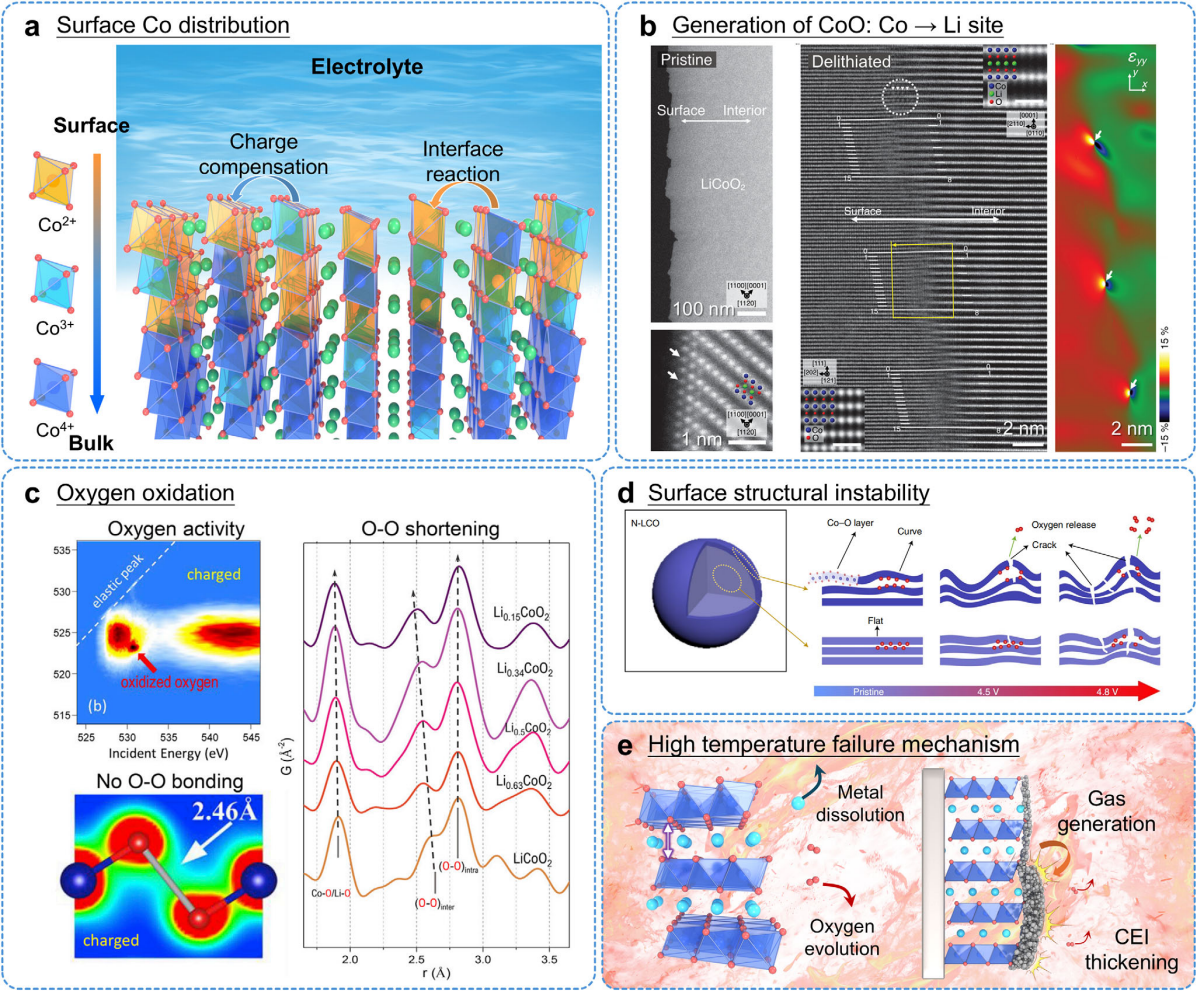
(a) Schematic illustration of the surface Co distribution under high‐voltage. (b) ADF STEM images of the pristine LCO sample. Atomic structural analysis of the interface between the outer CoO and inner LCO regions of the delithiated LCO sample. Reproduced with permission [67]. Copyright 2024, American Chemical Society. (c) Mapping of resonant inelastic X‐ray scattering of LCO at highly charged state. Sectional view of partial charge density within the energy spectrum of TM─O bonding or LCO at charged states. *Ex situ* neutron pair distribution function of LCO during the charging process. Reproduced with permission [69]. Copyright 2023, Cell Press. (d) Schematic illustration of the LCO structural evolutions during charge. Reproduced with permission [63]. Copyright 2021, Springer Nature. (e) High temperature failure mechanism.

Minimal Co dissolution or capacity degradation was detected when cycling at 4.2 V [65]. However, increasing the charging voltage to 4.5 V resulted in substantial Co dissolution, demonstrating a clear correlation between elevated voltage, Co dissolution, and accelerated capacity fading, as shown in Figure 6a. According to current understanding, high‐voltage operation induces corrosive attack on cathode particle surfaces, which subsequently triggers TM ion dissolution and disturbs interfacial processes at both cathode and anode [66]. ADF‐STEM characterization (Figure 6b) confirms that the cathode material undergoes stepwise structural evolution under deep delithiation [67]. First, Co ions migrate into Li layers, initiating the formation of a CoO‐like phase. This phase transition subsequently generates nanoscale porosity and lattice expansion, ultimately creating interfacial misfit dislocations between the newly formed CoO domains and the original layered structure. The shuttle effect transports dissolved transition metal cations from the cathode to the anode, where their deposition interferes with Li^+^ ion intercalation and deintercalation processes, slowing down overall Li^+^ transport kinetics [68].

With prolonged cycling, Co dissolution promotes the formation of a CEI layer, creates localized corrosion zones, and increases surface resistance. This direct dissolution of Co from the lattice reduces the amount of electrochemically active material in LCO, representing a major contributor to rapid capacity fade. Various surface byproducts further impede Li^+^ ion mobility. Under high‐voltage conditions, extraction of Li^+^ ions partially oxidize Co^3+^ to Co^4+^ to maintain charge neutrality. These highly reactive Co^4+^ species then interact with surface peroxides, accelerating reconstruction of the cathode surface. Simultaneously, electrolyte decomposition at high potentials enhances Co dissolution, typically manifested by rapid capacity loss during initial high‐voltage cycles. Immediately upon contact between LCO and electrolyte under high voltage, surface Co^3+^ ions undergo rapid reduction to Co^2+^. Elevated charge voltages amplify localized structural distortions at the cathode surface, with these defects progressively extending into the bulk material [64]. This underscores the dual necessity of developing robust high‐voltage cathode materials and minimizing electrode‐electrolyte interfacial exposure. Such strategic optimization simultaneously improves surface structural integrity and suppresses parasitic side reactions.

Oxygen release from LCO cathodes directly reflects surface structural breakdown and serves as a primary trigger for thermal instability in cathode materials [70]. The oxygen evolution reaction (OER) becomes activated only when a specific overpotential is reached above approximately 4.3 V. When LIBs are charged beyond 4.5 V during delithiation, structural instability develops through two interconnected processes: (1) the surface layered framework loses stability as excessive Li^+^ ions are removed, and (2) lattice oxygen becomes susceptible to oxidation and gaseous release. Earlier studies have established that oxygen evolution in layered oxides originates from the hybridization between oxygen 2p and TM 3d orbitals under high‐voltage conditions [71]. The voltage threshold for significant oxygen release lies at 4.5 V, beyond which the lattice oxygen in cathode materials becomes thermodynamically unstable. Integrated analysis using X‐ray spectroscopy, scattering techniques (X‐ray and neutron), and computational modeling has clarified the oxygen redox behavior in LCO, revealing oxidation of bulk lattice oxygen without localized O─O dimerization—suggesting limited oxygen release from the bulk material (Figure 6c) [69]. Importantly, interfacial reactions between LCO and electrolyte at high voltages drive surface degradation (Figure 6d), where structural defects initiate oxygen evolution through surface‐specific pathways [63]. Oxygen loss triggers collapse of the cathode surface architecture and accelerates electrolyte oxidation. These degradation processes are significantly accelerated at elevated temperatures (>60°C), leading to severe battery failure. This failure results from the combined effects of metal ion dissolution, lattice oxygen release, and continuous side reactions with the electrolyte (Figure 6e).

Extended cycling experiments conducted by Zhou et al. revealed the development of physical voids associated with localized oxygen vacancy formation, indicating surface peroxide generation and lattice oxygen release. While the detailed mechanisms of oxygen redox chemistry (including charge transfer pathways) remain incompletely characterized [72], researchers generally agree that high‐voltage‐induced peroxide formation and oxygen release progressively damage the LCO surface structure. This degradation process gradually converts the original layered arrangement into disordered spinel and rock‐salt phases [73]. These structural changes elevate interfacial charge transfer resistance and accelerate capacity fading. Furthermore, continuous oxidation of unstable lattice oxygen in high‐valence states weakens Co─O covalent bonding, generating under‐coordinated oxygen species. Therefore, preserving lattice oxygen stability at the cathode‐electrolyte interface stands as a crucial design objective for both high‐voltage LIBs and high‐temperature applications.

**FIGURE 7:**
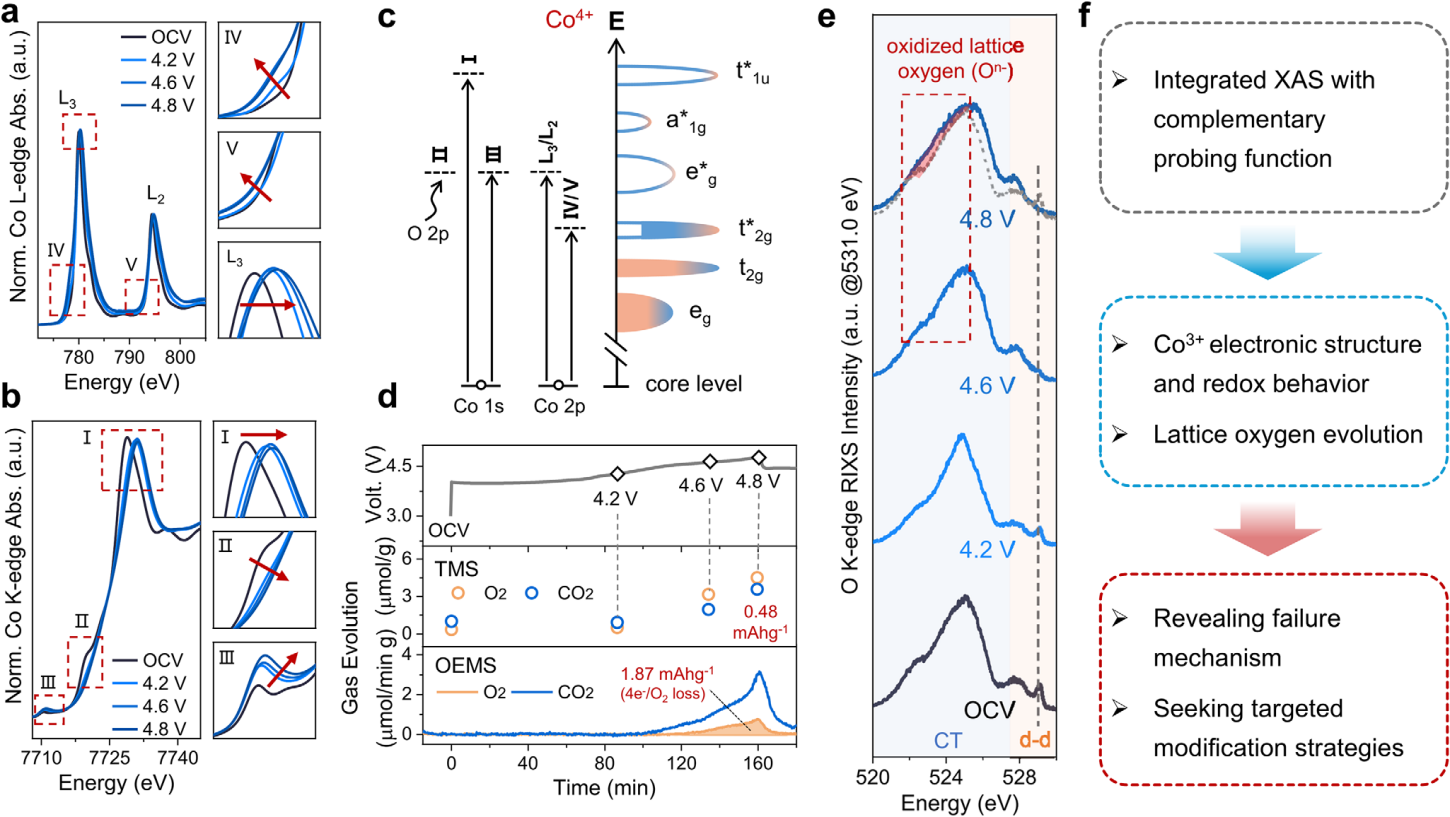
(a) *Co‐L_3_* edge XAS spectra. (b) *O‐K* edge XAS spectra. (c) *Co‐K* edge/*L_3_* edge XAS result and corresponding electronic configurations of Co^4+^. (d) Gas generation results for LCO. (e) Resonant inelastic X‐ray scattering of *O‐K* edge. (f) The workflow diagram of integrated XAS with complementary probing function. Reproduced with permission [74]. Copyright 2023, American Chemical Society.

Additionally, under high‐voltage conditions, Co dissolution and oxygen loss act together to accelerate localized structural breakdown in LCO cathodes. This synergistic degradation ultimately produces isolated corrosion zones near the particle surface. X‐ray absorption spectroscopy (XAS) has emerged as a powerful technique for investigating the operational mechanisms of rechargeable batteries, offering essential information about failure processes under high voltage, interfacial reactions between electrodes and electrolytes, and improvements achieved through surface modifications [75, 76, 77]. As show in Figures 7a–c, XAS has proven highly effective for monitoring dynamic variations in the electronic structure and redox behavior of cathode materials during electrochemical cycling. By analyzing the *O‐K* edge and gas generation (Figures 7d–f), researchers can directly track the evolution of lattice oxygen. Similarly, the *Co‐K* edge provides information on changes in the local coordination environment of cobalt atoms. XAS measurements confirm the oxidation of Co^3+^ to Co^4+^ when charged to 4.8 V. A new feature appeared near 524 eV in the *O‐K* edge spectrum at 4.8 V, which is associated with transitions to unoccupied O 2p states. Such transitions only occur when O^2−^ undergoes oxidation. To more directly visualize structural transformations in LCO during charging, TEM was used to examine samples at different delithiation states. Under deep charging conditions, a fraction of Co ions migrates into Li layers, promoting the development of spinel and rock‐salt phases. The surface region of LCO showed non‐uniform distribution of Li and Co, along with the appearance of an irreversible Co_3_O_4_ [78]. These findings support the conclusion that rapid capacity decline in LCO under high voltage connects directly to the irreversible formation of spinel phases on the cathode surface. Regarding redox reactions involving O^2−^, irreversible Co migration causes voltage hysteresis between charging and discharging processes, matching electrochemical observations of this phenomenon. Consequently, suppressing irreversible TM ion migration becomes essential for minimizing voltage hysteresis effects in disordered rock salt cathode materials.

### 3.3 Electrolyte Oxidation and Surface Degradation

For LIBs, the electrolyte functions as an ionic conduit between electrodes, enabling Li^+^ ion transport across the separator while maintaining electronic insulation. A fundamental understanding of electrolyte behavior and degradation pathways is essential for enhancing LIB cycling stability. Electrolyte failure mechanisms primarily involve: (1) formation of new interfacial layers at electrode‐electrolyte interfaces, (2) oxidative decomposition under high‐voltage operation, and (3) performance deterioration caused by electrolyte breakdown products. These processes critically influence battery lifespan and performance [74]. Elucidating the underlying principles of electrolyte oxidation and decomposition is therefore vital for developing stable high‐voltage electrolytes [79].

During extended cycling of LIBs, oxidation and reduction reactions occur at electrode‐electrolyte interfaces, resulting in the formation of interphase layers. Conventional electrolytes typically demonstrate adequate stability under normal operating conditions. However, as the charging cut‐off voltage increases, these standard commercial electrolytes begin to exhibit significant instability issues. Electrolyte stability is determined not only by the redox potential of the solvent components but also by their molecular interactions with other solvent species, electrolyte salts, and electrode surfaces. The degradation mechanism of electrolytes represents a complex process involving multiple contributing factors. Among these, the dissolution of TM ions and their subsequent catalytic reactions with electrolyte components substantially accelerate electrolyte failure [80]. When charging potentials exceed 4.3 V, layered transition metal oxide cathodes catalyze oxidative decomposition of the electrolyte at their surfaces, initiating parasitic degradation pathways. At elevated voltages, organic constituents such as EMC and EC solvents undergo increasingly severe oxidative decomposition reactions with lithium salts like LiPF_6_. This intensified decomposition leads to the formation of thick CEI films alongside gas evolution [81, 82].

**FIGURE 8:**
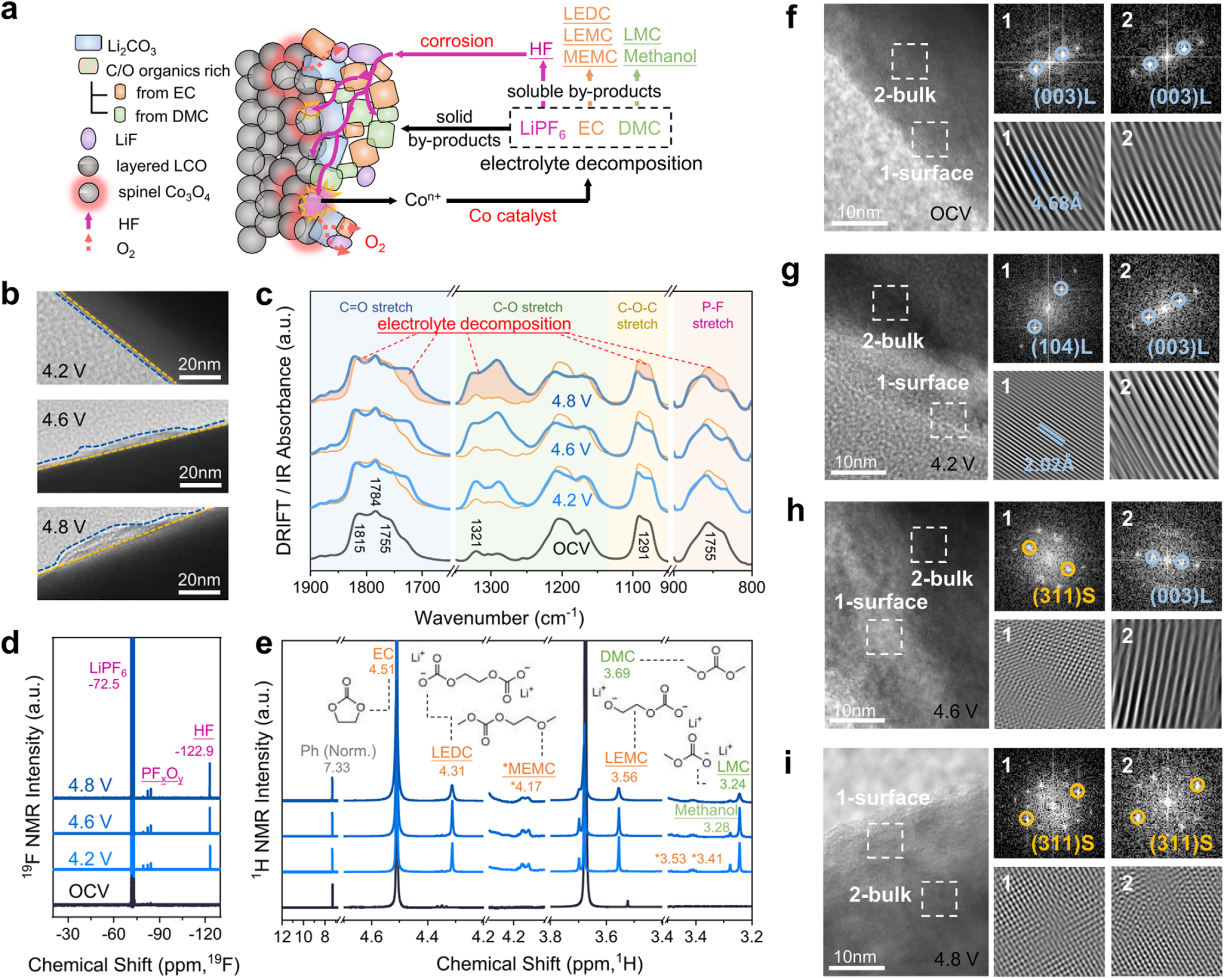
(a) Scheme of CEI formation, electrolyte decomposition, and LCO surface reconstruction. (b) CEI of the above cathodes. (c) DRIFT spectrum of the pristine electrolyte, 4.2, 4.6, and 4.8 V cathode surfaces obtained from the first cycle. (d) ^19^F and (e) ^1^H NMR spectra of pristine electrolyte, and three samples after 100 cycles from 3.0 to 4.2, 4.6, and 4.8 V. HR‐TEM images for (f) pristine LCO and cathodes after 50 cycles from 3.0 to (g) 4.2, (h) 4.6, and (i) 4.8 V and the corresponding FFT images and IFFT images. Reproduced with permission [74]. Copyright 2023, American Chemical Society.

As shown in Figure 8a, increasing voltage accelerates parasitic side reactions and byproduct deposition, while simultaneously activating compensatory decomposition pathways. Notably, oxidative oxygen loss from the lattice promotes formation of electrochemically inactive Co_3_O_4_ domains that impede Li^+^ diffusion, directly contributing to capacity deterioration. Furthermore, TEM analysis (Figure 8b) reveals the concurrent development of an unstable CEI, exacerbating interfacial degradation. The diffuse reflectance infrared Fourier transform (DRIFT) spectra (Figure 8c) reveal spectral changes indicative of the hydrolysis of DMC and LiPF_6_. These spectral features exhibit larger shifts with increasing delithiation, suggesting more severe electrolyte decomposition. Nuclear magnetic resonance (NMR) spectra (Figures 8d,e) are utilized to investigate soluble species in detail. Furthermore, the LCO maintains its layered structure (Figure 8f) after cycling between 3.0 and 4.2 V (Figure 8g). However, upon charged to 4.6 V, a Co_3_O_4_‐like spinel phase appears on the surface (Figure 8h) and subsequently within the bulk at 4.8 V (Figure 8i).

During prolonged cycling of the cathode, continuous oxidative decomposition reactions repeatedly expose fresh LCO surfaces to the electrolyte, creating a self‐sustaining cycle of cathode degradation. This process involves substantial Co dissolution, and the resulting Co ions exhibit significant catalytic activity under high‐voltage conditions, which further accelerates electrolyte decomposition [83]. Intensive electrolyte oxidation at elevated voltages generates abundant decomposition products on the cathode surface, increasing interfacial resistance and hindering Li^+^ ion transport. Additionally, dissolved Co species migrate through the electrolyte and deposit on the anode side, where they damage the initially formed solid electrolyte interphase (SEI) and promote further electrolyte decomposition [84, 85]. To address this challenge, future efforts should focus on developing more stable electrolyte formulations and novel functional additives capable of suppressing both TM ions dissolution and its catalytic effects. Such advancements would help minimize electrolyte decomposition and extend the cycle life of high‐voltage batteries.

Substantial experimental evidence confirms that electrolyte oxidation and the accompanying growth of the CEI layer under high‐voltage conditions significantly slow Li^+^ ion diffusion kinetics. Meanwhile, the persistent oxidized state of the cathode surface and the continual exposure of fresh interfacial areas further accelerate electrode degradation, ultimately leading to more severe capacity loss [86]. While the CEI layer increases interfacial resistance and hinders Li^+^ mobility, its in situ formation provides critical protection by physically isolating the cathode surface from the electrolyte. This barrier effect substantially mitigates further interfacial degradation through the suppression of continuous electrolyte oxidation and limitation of transition metal dissolution. Nevertheless, inherent thermodynamic instability makes certain degree of organic electrolyte decomposition unavoidable under high‐voltage operation.

Therefore, establishing an effective CEI layer becomes essential to protect the cathode surface, minimize interfacial resistance, and facilitate Li^+^ ion transport. One practical approach involves incorporating functional additives into the electrolyte, which participate in electrochemical reactions during cycling to form a durable and uniform CEI coating. This engineered interfacial layer serves a dual‐purpose: it shields the cathode from parasitic reactions while promoting efficient Li^+^/electron transfer. As a result, cells employing such additives demonstrate markedly enhanced cycling performance under high‐voltage conditions. These insights substantially advance our understanding of interfacial electrochemistry and support the rational development of improved electrolyte systems and surface modification strategies, ultimately contributing to longer‐lasting and safer battery technologies.

When engineering new LIB systems, designers must account for stable voltage of the cathode material and current collector, while also ensuring compatibility with the electrochemical stability window of the electrolyte. This operational voltage range is fundamentally governed by the energy gap between its highest occupied (HOMO) and lowest unoccupied (LUMO) molecular orbitals [87]. Operating outside this stable voltage window triggers decomposition processes: at the anode, potentials below the LUMO level cause electrolyte reduction, whereas at the cathode, potentials above the HOMO level induce electrolyte oxidation. In practice, the SEI on the anode and the CEI on the cathode can partially mitigate these side reactions. Consequently, developing thin, stable, and intrinsically protective SEI and CEI films has become a major research priority. Designing these thin, intrinsic protective layers and studying their protective mechanisms are pivotal for the advancement of high‐voltage batteries.

## 4 Enhancement Strategies of High‐Voltage LCO

The previous sections have provided a systematic examination of degradation mechanisms in LCO cathodes and LIB systems under high‐voltage operation. While increasing the charge cutoff voltage effectively improves capacity, it also introduces complex instability issues. To address these challenges, recent scientific efforts have concentrated on developing durable LCO cathodes capable of maintaining structural and electrochemical stability at elevated voltages. Considerable advancements have been achieved through innovative modification approaches focused on interface stabilization and bulk structural preservation. Currently, prevailing enhancement strategies primarily include foreign‐ion doping, surface coating, particle architecture design, and electrolyte engineering. This section aims to provide a comprehensive overview of these strategies, discussing their respective advantages and current research developments.

### 4.1 Foreign‐ion Doping

Foreign‐ion doping has become a widely adopted method for tuning the crystal and electronic structures of layered cathode materials, with substantial research progress reported in recent years. Doping of selected elements enables precise control over various physical properties of cathode materials, including electronic structure, bandgap characteristics, charge distribution, and lattice parameters. Substitutional doping at TM sites has been effectively utilized to enhance electrical conductivity and elevate electrochemical potential. Simultaneously, doping at Li sites creates structural pillars that maintain interlayer spacing while enabling fast Li^+^ ion migration, thereby improving both mechanical stability and charge transport kinetics. Achieving optimal doping performance requires careful optimization of several key parameters: dopant selection, doping concentration, doping sites, and doping methods.

**FIGURE 9:**
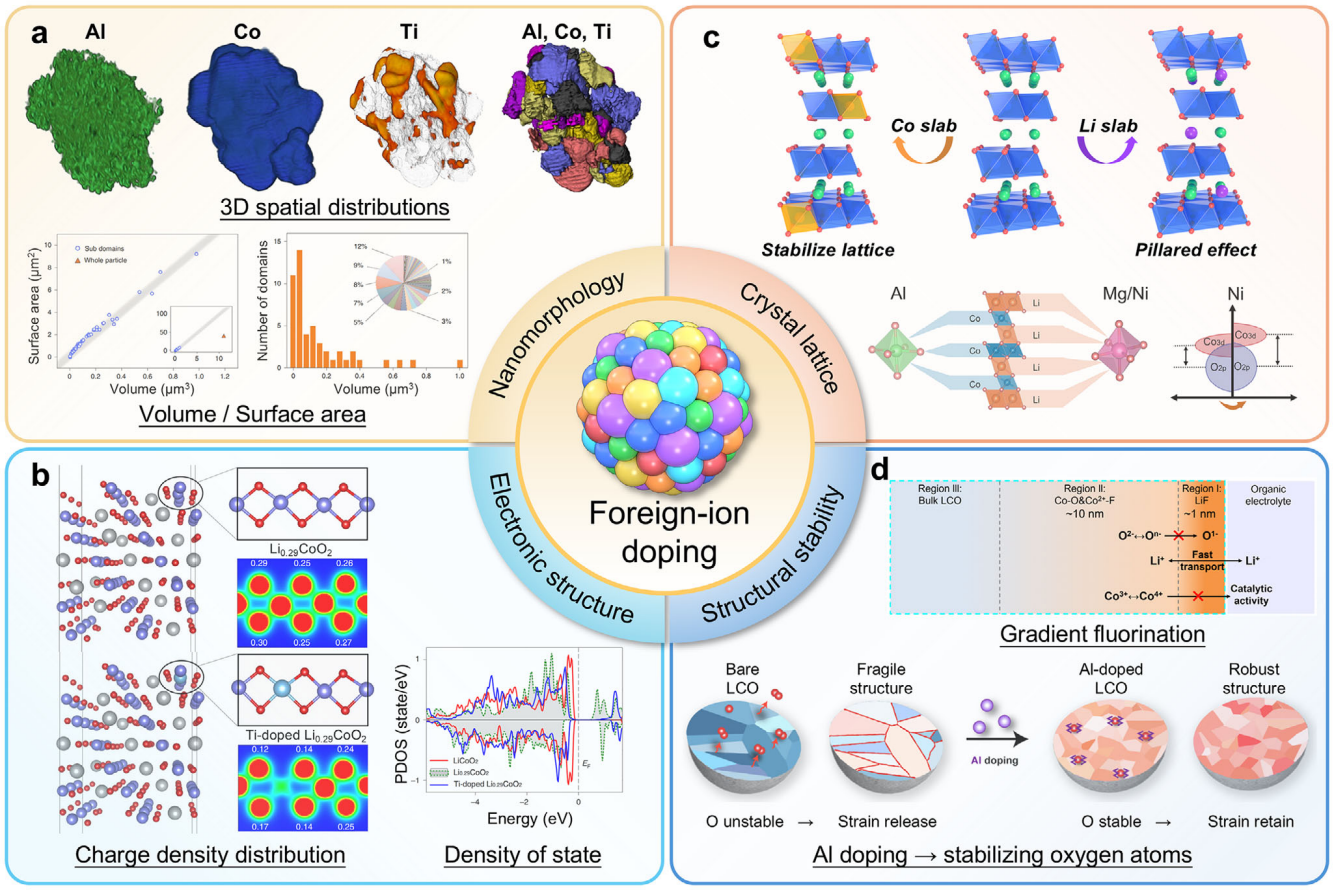
Advantages of the foreign‐ion doping for layered TM cathode. (a) Nanomorphology: distribution of Al, Co and Ti [88]. (b) Electronic structure: Charge density distribution and density of states (DOS) for Li_0.29_CoO_2_ and Ti‐doped Li_0.29_CoO_2_. Reproduced with permission [88]. Copyright 2019, Springer Nature. (c) Crystal lattice: Doping into Co/Li slab; Al, Mg and Ni co‐doping. Reproduced with permission [89]. Copyright 2025, WILEY‐VCH. (d) Surface stability: Gradient fluorination. Reproduced with permission [92]. Copyright 2024, Royal Society of Chemistry; Serves as a stabilizing agent for oxygen atoms. Reproduced with permission [93]. Copyright 2025, American Chemical Society.

As shown in Figure 9, strategic elemental doping significantly enhances the cycling stability of cathode materials under high‐voltage conditions, primarily through four interconnected mechanisms: (1) Nanomorphology [88]: regulating the spatial distribution of dopants to achieve tailored nanostructural features with desired modification effects (Figure 9a). (2) Electronic structure [88]: modifying electronic band structures and charge distribution to improve bulk electronic conductivity and accelerate Li^+^ ion transport kinetics (Figure 9b). (3) Crystal lattice [89]: strengthening covalent bonding between dopant elements and oxygen atoms to stabilize lattice oxygen and suppress irreversible oxygen redox activity, while simultaneously optimizing interlayer spacing to facilitate Li^+^ ion diffusion (Figure 9c). (4) Surface stability [90, 91, 92, 93]: inhibiting irreversible phase transitions at high voltages, thereby mitigating bulk volume variations and releasing mechanical stress (Figure 9d).

One of the earliest doping strategies for LCO is the doping of TM elements, as the LiTMO_2_ family includes many classical cathode materials such as LiNiO_2_, LiMnO_2_, LiAlO_2_, and LiFeO_2_ [94, 95, 96, 97, 98]. As the dopant concentration increases, a significant challenge arises regarding the ability of industrially scalable synthesis methods to achieve uniform incorporation of dopant elements into the cathode oxide lattice [99, 100, 101]. For example, Duffiet et al. observed chemical inhomogeneity in Al‐doped LCO cathodes, noting the formation of Al‐rich domains even at a moderate doping level of 4 at%, despite the absence of long‐range phase separation [102]. This inhomogeneous distribution of chemical elements can lead to various types of structural defects within the material, including dislocations, stacking faults, twin boundaries, antiphase boundaries, and domain boundaries, which deviate from the ideal single‐crystal layered structure [103, 104]. Furthermore, for compact portable electronics that require smaller battery dimensions while delivering higher energy density, the conventional LCO single crystal (with its inherently high volumetric energy density) remains the most suitable candidate.

Introducing transition metal elements through doping creates a solid solution system with the general formula LiTM*_x_* Co_1−_ *_x_* O_2_ [39]. The strategic inclusion of cations such as Mg, Al, Ti, Ni, Mn, Fe, Zr, La and Ce allows systematic tuning of the electronic environment. This method concurrently improves high‐rate performance and long‐term cycling stability [105, 106]. The doping efficacy of these elements is fundamentally constrained by their solid solubility limits. High‐valence cations (Ti, Zr and Ce) exhibit preferential segregation at LCO particle surfaces rather than bulk incorporation. These dopants form exceptionally strong TM–O bonds that locally stabilize the oxygen sublattice, effectively suppressing surface oxygen evolution during high‐voltage operation. As for elements like Al, it has a similar ionic radius to Co. Elements such as Al, which have an ionic radius comparable to Co, are well‐suited for bulk substitution at TM sites. Al has consequently become one of the most widely adopted dopants for LCO [107]. Appropriate Al doping helps suppress Co dissolution and enhances the stability of the crystal structure. Recent advanced doping approaches, including La/Al co‐doping LCO [107], Se‐LCO [108], Mg‐LCO [109], Al‐F‐LCO [110], F‐LCO [92] and Mg, Al, Ti‐LCO [111], have consistently demonstrated that elemental doping reinforces the structural integrity of the cathode, thereby extending the cycle life of LCO under high‐voltage conditions.

As a representative example, Mg‐doped LCO offers several beneficial properties, including high structural stability, improved electrical conductivity, and a high melting point. Xie et al. have performed detailed investigations into the mechanisms underlying performance enhancement in Mg‐doped LCO [112]. Substituting Co with Mg increases the electrical conductivity of the material, leading to better reaction kinetics. In a complementary approach, Huang et al. have utilized Mg substitution for Li^+^ slab acts as an interlayer pillar to prevent structural collapse under deep delithiation [109]. The stable interlayer structure contributes to an improved diffusion rate of Li^+^ ions and suppresses the high‐voltage phase transition.

**FIGURE 10:**
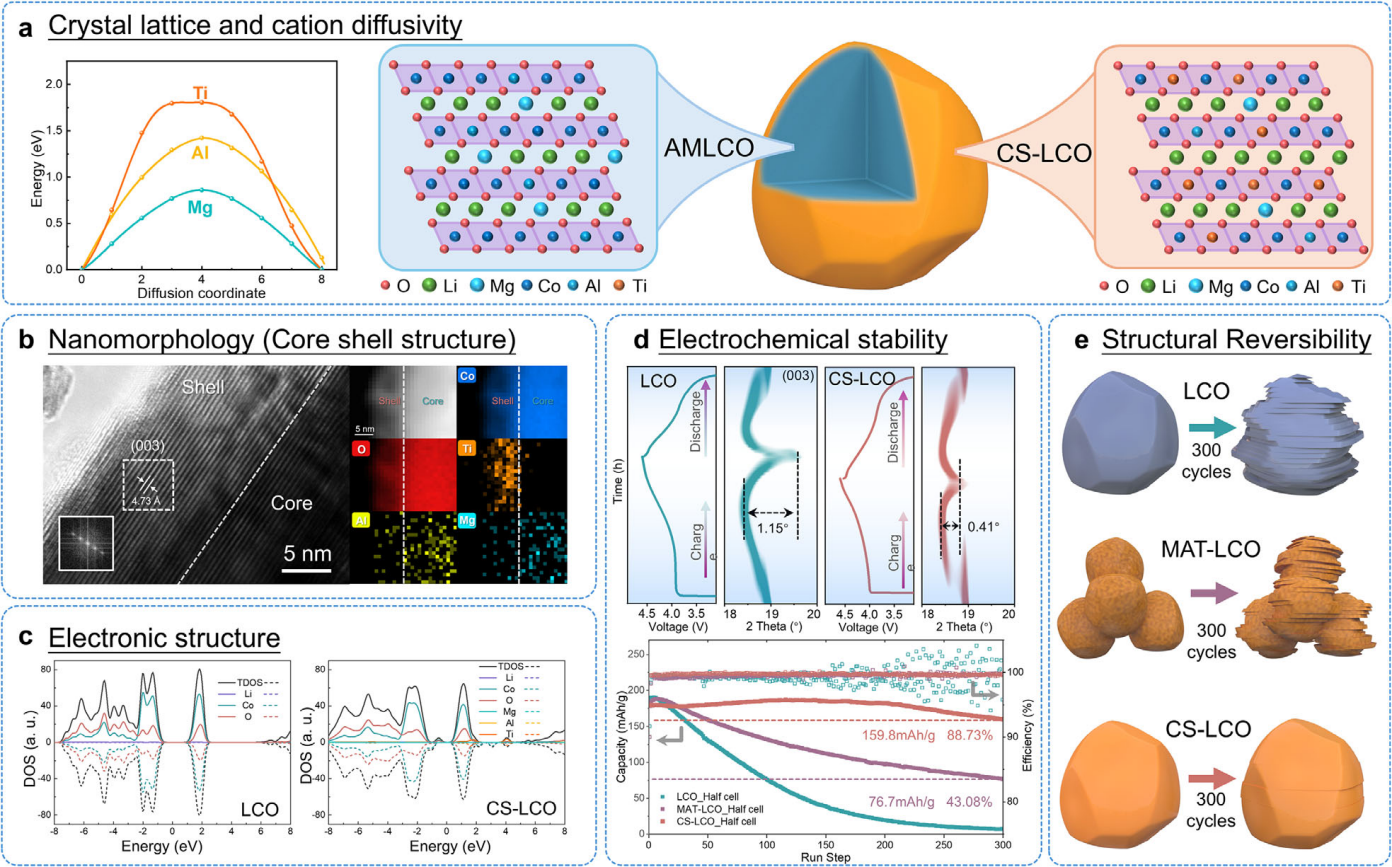
(a) Crystal lattice and cation diffusivity: DFT calculation of diffusion energy barrier of Mg, Al and Ti ions along LCO interlayer; Structural design of core‐shell LCO with Al/Mg bulk co‐doping and gradient surface Ti doping. (b) Nanomorphology: HRTEM image of CS‐LCO. Inset indicates the FFT pattern of white rectangle; Elemental distribution of O, Co, Ti, Al and Mg near the surface. (c) Electronic structure: DOS of CS‐LCO. (d) Electrochemical stability: Charge/discharge curves and in situ XRD results of LCO and CS‐LCO; Cycling stability of half‐cells at 1C under 4.6 V from the first to 300^th^ cycles. (e) Schematic diagrams of the structural evolution after long‐time cycling. Reproduced under terms of the CC‐BY Creative Commons Attribution 4.0 International License [111]. Copyright 2024, Zezhou Lin, published by Springer Nature.

In summary, trace‐level doping has become the predominant strategy for enhancing LCO cycling durability while preserving its fundamental crystal framework [86]. Multi‐element doping offers additional performance benefits by combining the complementary functions of different dopant species. Our research group has developed a kinetic‐controlled two‐step doping method to prepare core shell LCO (CS‐LCO) that spatially arranges dopants according to their respective diffusion coefficients (Figure 10) [111]. This approach enables high‐mobility Al^3+^/Mg^2+^ ions to distribute uniformly throughout the grain core, while low‐mobility Ti^4+^ ions preferentially accumulate at the particle surface. In situ XRD analysis confirms that this graded doping configuration alleviates *c*‐axis strain and effectively inhibits structural degradation mechanisms. When cycled at a high cut‐off voltage of 4.6 V, the resulting single‐crystal core‐shell LCO cathode delivers high reversible capacity with excellent capacity retention of approximately 89% after 300 cycles. This design concept can be extended to other cation pairs combining low‐diffusivity species (Zr, W, and Ta, etc.) with high‐diffusivity ions (Zn, Fe and Ni, etc.). This generalizable design principle enables rational engineering of other layered oxide cathodes through selective cation pairing, opening new avenues for high‐voltage battery development.

Rational multi‐element doping therefore represents a highly promising approach to developing structurally stable LCO cathodes under high‐voltage operation. However, current practices for dopant selection, concentration optimization, and site‐specific incorporation still depend heavily on experimental findings. The underlying mechanisms and precise structure‐activity relationships require further clarification. Moreover, while elemental doping improves the overall electrode structure, the doping process itself demands strict synthetic control, including high‐temperature annealing above 800°C and accurate Li source compensation. These requirements inevitably increase the complexity and cost of large‐scale manufacturing.

### 4.2 Surface Modification

Surface modification has become a widely implemented and effective approach to protecting cathode materials [113, 114]. As discussed earlier, volumetric changes and electrolyte decomposition at high voltages significantly contribute to cathode degradation. Applying a customized protective layer on the cathode surface reduces direct contact between the reactive electrode and the corrosive electrolyte. This protective measure not only suppresses undesirable interfacial reactions but also helps maintain the structural stability of LCO during high‐voltage cycling.

A well‐designed surface coating can deliver multiple functional advantages: (1) Electrochemical isolation: The coating acts as a physical barrier that limits electrode‐electrolyte contact, thereby reducing parasitic side reactions; (2) Interfacial kinetics enhancement: It facilitates Li^+^ ion transport and improves charge transfer efficiency across the interface; (3) Mechanical reinforcement: The protective layer increases the cathode's resistance to stress‐induced fracture; (4) Voltage tolerance: It stabilizes the electrode surface against oxidative breakdown at high operating potentials.

Previous research has extensively explored metal oxide matrices as coating materials for LCO cathodes due to their favorable compatibility. Materials such as Al_2_O_3_, ZrO_2_, LiMn_2_O_4_, TiO_2_ and ZnO have been widely investigated for their protective capabilities [115, 116]. However, the detailed mechanisms through which these coatings provide protection remain incompletely understood. Although coatings function as physical barriers to the cathode surface, they inevitably lead to some capacity reduction due to their electrochemically inactive nature. Under high‐voltage conditions, achieving an optimal balance between reversible capacity and long‐term cycling stability becomes crucial. This balance depends significantly on both the crystalline structure and the thickness of the coating layer. While amorphous coatings can provide effective protection, excessive thickness adversely affects battery performance. Overly thick coating layers impede Li^+^ ion transport across the interface, increase charge transfer resistance, and ultimately diminish reversible capacity due to slowed Li^+^ ion kinetics [117, 118, 119, 120].

**FIGURE 11:**
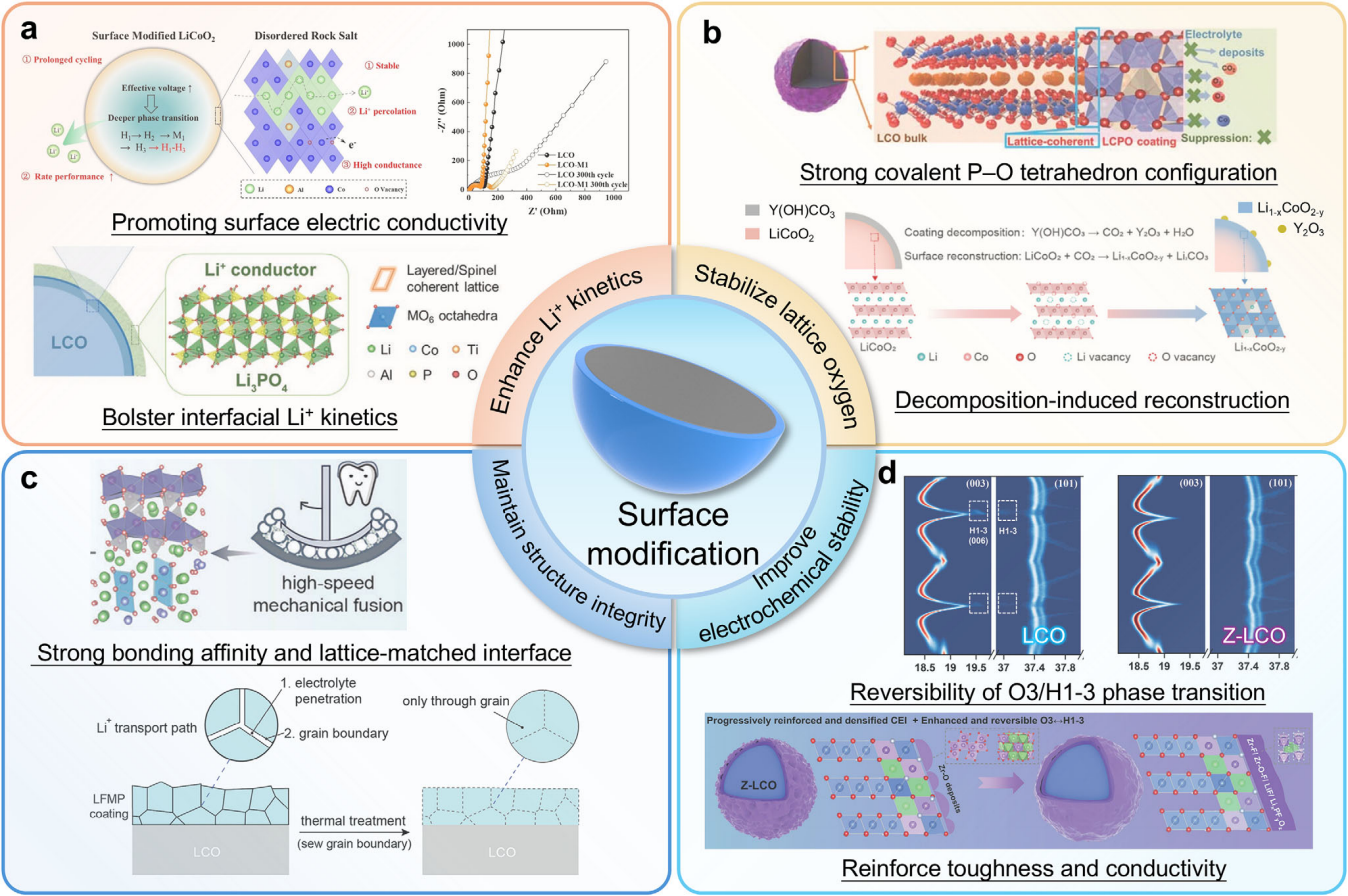
Advantages of surface modifications for cathode materials. (a) Enhance Li^+^ kinetics: promoting surface electric conductivity. Reproduced with permission [121]. Copyright 2023, WILEY‐VCH; Bolster interfacial kinetics. Reproduced with permission [122]. Copyright 2023, WILEY‐VCH. (b) Stabilize lattice oxygen. Reproduced with permission [123]. Copyright 2022, WILEY‐VCH. Reproduced with permission [124]. Copyright 2026, WILEY‐VCH. (c) Maintain structure integrity. Reproduced with permission [125]. Copyright 2024, WILEY‐VCH. (d) Improve electrochemical stability. Reproduced with permission [126]. Copyright 2024, WILEY‐VCH.

Recent research has focused extensively on developing advanced coating systems with targeted functions, including: enhanced Li^+^ kinetic (Figure 11a) [121, 127], suppressed side reaction (Figure 11b) [123], enhanced mechanical properties (Figure 11c) [125] and increased electrochemical stability (Figure 11d) [126]. Among the successfully demonstrated coating materials are Li─Al─F coating [127], Al_2_O_3_ [128], Li_1.5_Al_0.5_Ti_1.5_(PO_4_)_3_ [122], olivine enamel layer [125], LiNbO_3_ [129], and LiAlO_2_ coating [130]. While conventional metal oxide coatings provide surface protection, many suffer from limited Li^+^ ion conductivity, leading to increased interfacial resistance. This elevated resistance presents challenges for overall Li^+^ ion transport within the cathode system. Consequently, materials with high ionic conductivity have emerged as promising alternative coating candidates [131]. A novel Li_1.5_Al_0.5_Ti_1.5_(PO_4_)_3_ coating was recently developed for LCO through an in situ formation [122]. This Li^+^ conductive layer consists of Li_3_PO_4_ and Li_1.5_Al_0.5_Ti_1.5_(PO_4_)_3_ phases, which form a continuous ion‐conducting network across the LCO surface. This architecture not only enhances interfacial Li^+^ ion transport but also substantially improves cycling retention and rate capability. By effectively mitigating cathode‐electrolyte interface degradation, these phases work together to enhance both long‐term cycle life and high‐power performance of the electrode.

Surface coating layers play a crucial role in enhancing cathode performance that extends beyond simple physical protection. The interaction between the coating and the cathode surface promotes the migration of interfacial electrons and ions, which is essential for maintaining cycling stability under high‐voltage conditions [132, 133, 134]. Yan et al. developed an innovative high‐speed mechanical fusion technique to create a seamless, uniform enamel‐like olivine (LiFe_0.4_Mn_0.6_PO_4_, LFMP) coating on LCO particles (LFMP@LCO). This multifunctional coating strategy operates through several mechanisms: (1) From the outside inward, the LFMP enamel layer controls electrolyte decomposition to form a stable CEI, thereby suppressing Co dissolution and electrolyte breakdown products. (2) From the inside outward, the strong adhesion between LCO and LFMP stabilizes the oxygen framework and prevents formation of the harmful spinel Co_3_O_4_ phase. Additionally, (3) the LFMP olivine coating significantly improves the thermal stability of highly delithiated LCO by inhibiting the detrimental layered–to–spinel phase transformation and oxygen release at high voltages. As a result, the LFMP‐coated LCO cathode demonstrates exceptional cycling stability, retaining over 85% of its capacity after 200 cycles. This high‐speed solid‐phase coating method not only offers industrial scalability but also represents an innovative approach to stabilizing the surface and interface chemistry of high‐voltage LCO cathodes.

Surface modification serves as a highly efficient method for enhancing cathode stability, where precisely engineered coating architectures can substantially reduce degradation mechanisms in high‐voltage LCO systems. Nevertheless, several challenges in coating application require further investigation. For instance, solid‐state coating techniques may result in non‐uniform surface coverage. Solution‐based coating methods are only suitable for cathode materials that remain stable in aqueous environments. Other issues, such as achieving strong adhesion between the coating and cathode during high‐temperature annealing and managing Li loss, also need to be resolved. The layer quality, crystal structure, and thickness of the coating yield a substantial influence on enhancing the electrochemical performance of cathode, and these aspects require systematic research to gain a deeper understanding of their relationships.

Phosphate‐based coatings have recently gained attention as a promising strategy for achieving long‐term cycling stability. However, conventional amorphous coatings often suffer from limited Li^+^ conductivity due to disordered structures, ultimately restricting rate performance. An ideal coating architecture should simultaneously fulfill three critical requirements: (1) High crystallinity to facilitate rapid Li^+^ diffusion, (2) Tailored nano porosity to enable efficient Li^+^ desolvation at the interface, and (3) Conformal coverage to ensure complete cathode protection. This is yet very challenging.

**FIGURE 12:**
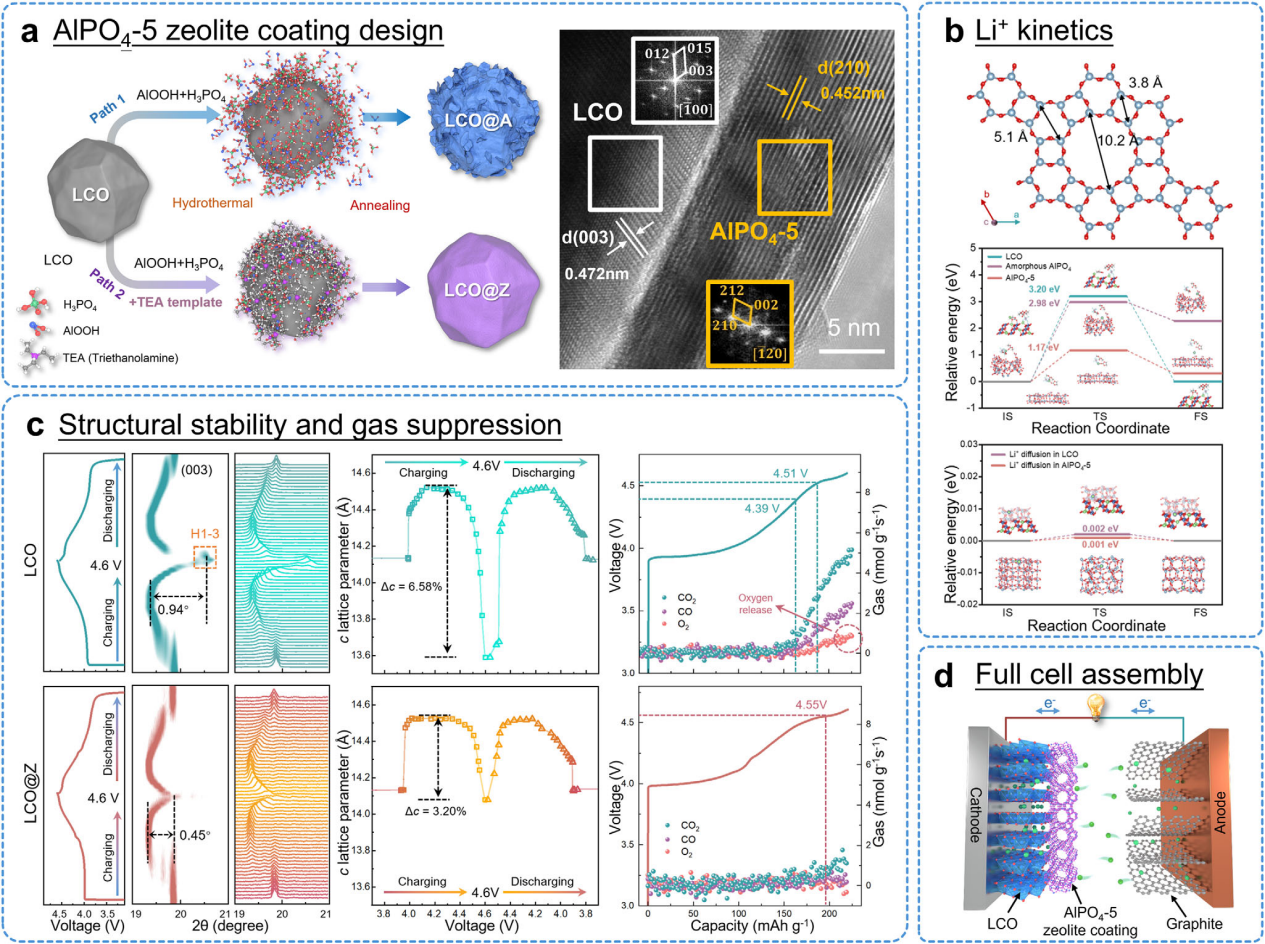
(a) Schematic illustration of the synthesis design of AlPO_4_‐5 zeolite coated LCO (LCO@Z); HRTEM images of LCO@Z. (b) Li^+^ kinetics. Lattice structure of AlPO_4_‐5 zeolite. DFT‐calculated energy barriers for the Li^+^ desolvation and the Li^+^ diffusion on AlPO_4_‐5 zeolite. (c) Enhanced structural stability and suppression of gas evolution under 4.6 V of LCO and LCO@Z. (d) Schematic illustration of LCO@Z full cell. Reproduced with permission [135]. Copyright 2025, Royal Society of Chemistry.

In previous work, the triethylamine (TEA) template was used to induce a crystallization of amorphous AlPO_4_ into crystalline AlPO_4_‐5 zeolite, achieving complete conformal coverage on LCO surfaces (Figure 12a). AlPO_4_‐5 zeolite coating has multifunctional effects: (1) Interfacial stabilization: The electrochemically inert and mechanically robust AlPO_4_‐5 zeolite coating acts as a shield, preventing direct electrolyte‐LCO contact. This suppresses lattice oxygen loss, mitigates parasitic side reactions, and minimizes CEI buildup, thereby alleviating surface degradation. (2) Facilitated Li^+^ transport: The crystalline nanoporous framework of AlPO_4_‐5 provides fast Li^+^ diffusion channels. The appropriate pore size promotes efficient Li^+^ desolvation, enhancing interfacial kinetics (Figure 12b). (3) Mechanical reinforcement: The elastic, fully continuous coating inhibits strain‐induced O3→H1‐3 phase transitions, preventing microcrack formation and preserving structural integrity (Figure 12c). As a result, this multifunctional AlPO_4_‐5 zeolite coating enables highly stable operation of LCO cathodes under high‐voltage conditions. In LCO@Z||graphite full cells, a specific capacity of 164.1 mAh g^−1^ (84.1% retention) is maintained after 500 cycles, significantly outperforming unmodified LCO, which degrades to almost zero under the same conditions (Figure 12d). This zeolite templating strategy establishes a scalable and effective approach for producing high‐energy‐density LIBs with outstanding long‐term cycling performance.

### 4.3 Structural Design

To improve the electrochemical performance of LCO cathodes, elemental doping and surface coating utilize distinct mechanisms. In an effort to further extend the cycling stability of cathode materials under high‐voltage conditions, researchers have begun exploring structural design as a route to multifunctional enhancement, establishing a new frontier in cathode material development [136, 137, 138, 139, 140].

**FIGURE 13:**
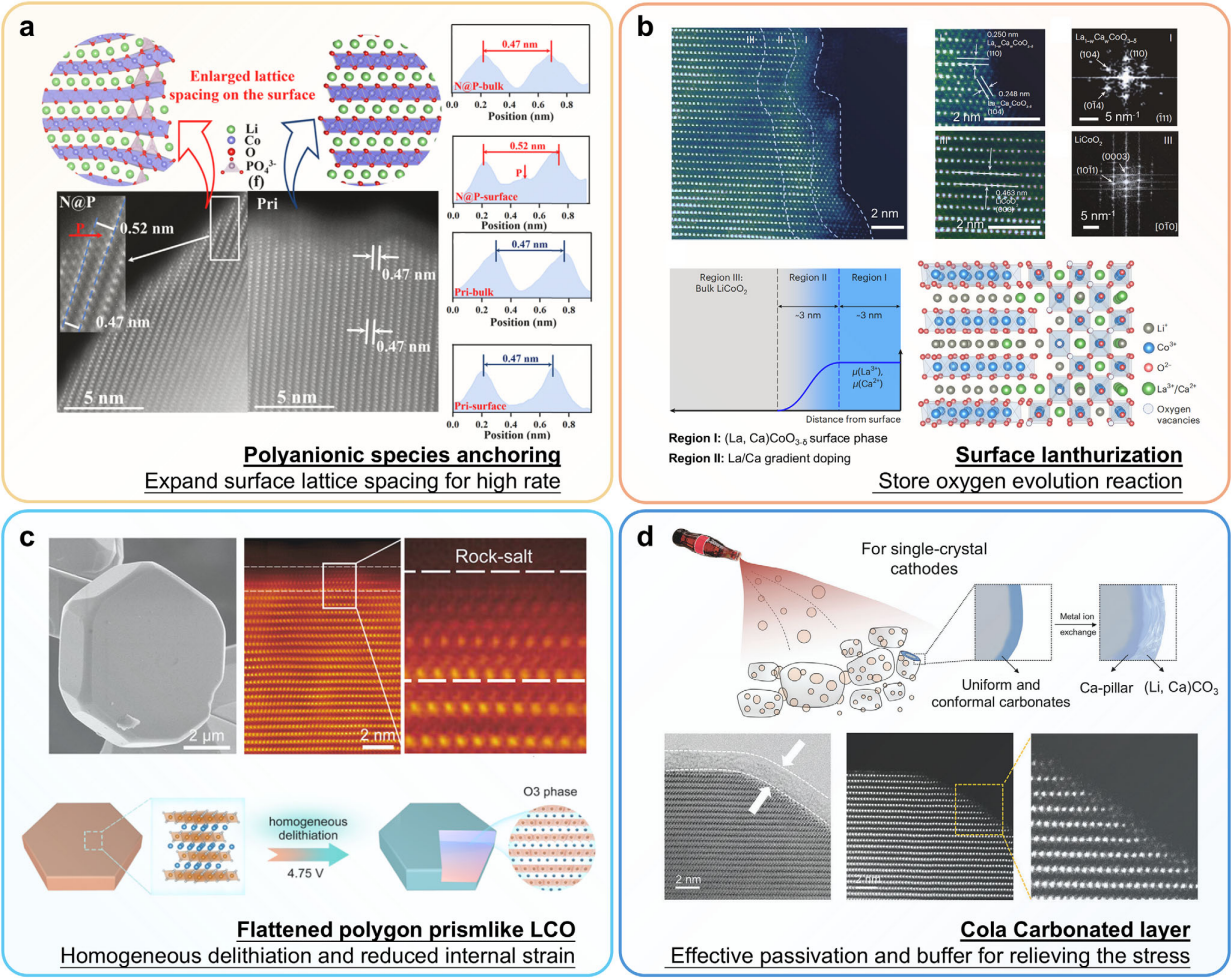
Recent research progress about structure design for high‐voltage LCO cathodes. (a) Anchored polyanionic (PO_4_)^3−^ species on surface. Reproduced with permission [141]. Copyright 2024, Royal Society of Chemistry. (b) Lanthurizing process to regulate the near‐surface structure. Reproduced with permission [142]. Copyright 2023, Springer Nature. (c) Flattened polygon prismlike LCO. Reproduced with permission [143]. Copyright 2025, American Chemical Society. (d) Cola carbonated layer for effective passivation. Reproduced with permission [144]. Copyright 2024, WILEY‐VCH.

One innovative approach involves the introduction of polyanionic species onto the LCO surface. These species function as “micro‐funnels” that widen the surface lattice spacing by approximately 10%, significantly accelerating Li^+^ ion diffusion and substantially improving rate capability. This modification leads to markedly enhanced reaction kinetics and structural stability [141] (Figure 13a). A separate advancement involves the development of a lanthurization process to strengthen the high‐voltage cycling endurance of LCO cathodes (Figure 13b) [142]. This method employs a uniform ion‐exchange mechanism that reconstructs the near‐surface region, forming a strained, high‐quality surface structure which reduces Li vacancy concentration and inhibits surface phase transformation.

Zhang et al. developed an innovative molten‐salt synthesis approach to engineering flattened polygonal prismatic LCO (P‐LCO) particles with unique structural advantages (Figure 13c) [143]. This universal morphology‐control strategy enables symmetrical *c*‐axis alignment that promotes uniform Li^+^ flux during (de)intercalation. The reaction homogeneity significantly improves bulk‐phase reaction kinetics throughout the particle. As a result, it mitigates anisotropic lattice strain at high‐voltages and suppresses the deleterious O1 phase transformation. Liao et al. devised a highly conformal and extensible interfacial modification method utilizing supersaturated CO_2_ bubbles to stabilize LCO cathodes at high voltages [144], achieving outstanding capacity retention and energy density (Figure 13d). This innovative carbonation strategy, drawing inspiration from carbonated beverage chemistry, resolves key interfacial challenges in high‐voltage cathodes and creates new possibilities for future high‐energy‐density LIBs. Another significant development involves engineering a surface rock‐salt layer on LCO (RS‐LCO) cathode material to enhance its structural durability at operating voltages up to 4.65 V [134]. This rock‐salt layer effectively suppresses surface oxygen release, minimizes side reactions, and retards structural degradation, while simultaneously preventing bulk phase separation and crack formation. The gradual phase transition from rock‐salt to an ionically conductive spinel phase contributes to a capacity activation process and improves the rate performance of the modified material.

**FIGURE 14:**
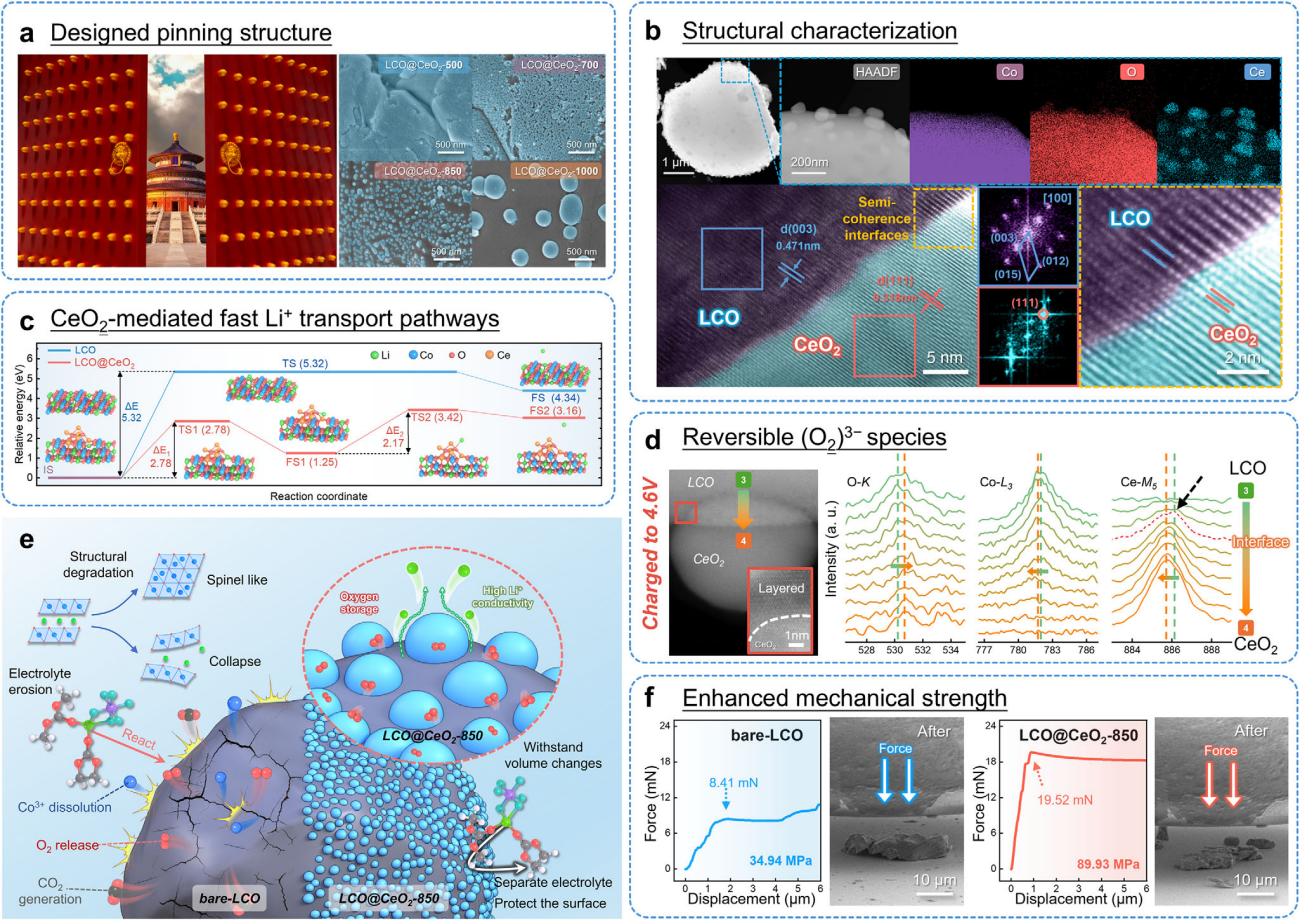
(a) Designed pinning structure. (b) Morphology characterization and element distribution of LCO@CeO_2_‐850. (c) CeO_2_‐mediated fast Li^+^ transport pathways. (d) Reversible (O_2_)^3−^ species testing by EELS. (e) Schematic illustration of the mechanism of the CeO_2_‐850 in enhancing the electrochemical performance. (f) Enhanced mechanical strength. Force‐displacement curves for indentation phases. Reproduced with permission [145]. Copyright 2026, WILEY‐VCH.

The structural design of the near‐surface region is crucial for achieving multifunctional effects in battery materials. This concept can be illustrated by an analogy with the golden nails on the vermilion gates of the Forbidden City. These nails were originally used to fasten wooden boards together, preventing the gate from splitting while maintaining its visual appeal. Inspired by this “pinning reinforcement” mechanism (Figure 14a), our team has developed a CeO_2_ nanoparticle pinning structure to improve the electrochemical stability of LCO cathodes at high cut‐off voltages of 4.6 V [145]. In this design, CeO_2_ nanoparticles act as “nano‐nails” that are uniformly anchored on the LCO surface (Figure 14b). This arrangement forms discrete but evenly distributed pinning sites. These sites help accommodate volume changes during cycling, reduce crack formation, and minimize the amount of inactive material. At the same time, the CeO_2_ pins create fast pathways for Li^+^ ion transport, which significantly improves the rate performance of the material (Figure 14c). Additionally, thanks to their oxygen vacancies and reversible Ce valence changes, these nanoparticles also serve as an “oxygen reservoir” under high voltage (Figure 14d). This function helps stabilize oxygen redox activity and suppress oxygen loss during charging and discharging. This design effectively buffers volume change while also enhancing structural stability under high stress conditions (Figures 14e,f). Moreover, this improved mechanical performance holds promise for applications in solid‐state battery cathodes, where high‐stress conditions during cell assembly are often required.

In a similar direction, Dong et al. proposed a dual‐modification strategy for LCO cathodes [146]. Their approach combines a Zr‐doped surface layer to stabilize the bulk structure with surface Li_2_Zr(PO_4_)_2_ (LZPO) nanoparticles to enhance interfacial kinetics. The LZPO nanoparticles help achieve uniform electrolyte contact and support the formation of a thin, stable CEI with low resistance. As a result, the modified LCO cathode maintains 94% of its capacity after 100 cycles, with a Coulombic efficiency of 99.9%, demonstrating excellent electrochemical stability. Furthermore, Sun et al. employed an atomically thin MXene layer for surface modification [147]. This multifunctional design reduces direct contact between the cathode surface and the organic electrolyte, suppresses detrimental phase transitions, and limits Co dissolution, thereby enabling more stable operation of LCO at high voltages. In a complementary strategy, Tian et al. created an integrated modification system combining Mn/La bulk co‐doping with a protective Li─Ti─O surface coating on LCO [148]. This dual‐approach modification resulted in comprehensive improvement in the electrochemical performance of LCO. The bulk Mn/La co‐doping enhanced the stability of the crystal framework and improved Li^+^ ion transport kinetics. Simultaneously, the Li─Ti─O coating provided additional surface stabilization by isolating the cathode from electrolyte‐induced degradation. Although these multifunctional structural strategies operate through different mechanisms, they share the common objective of enhancing the electrochemical performance of LCO cathodes and achieving superior cycling stability under high‐voltage conditions. These innovative structural design approaches create new opportunities for advancing cathode material performance, though further investigation is required to fully understand their underlying mechanisms and optimize their implementation.

### 4.4 Electrolyte Optimization

The operation of LIBs with high‐voltage LCO cathodes often induces instability in conventional electrolytes, presenting a major obstacle for developing high‐energy‐density battery systems [34, 49]. When charged above 4.3 V, standard electrolytes containing LiPF_6_ salt and carbonate solvents undergo oxidative decomposition catalyzed by the LCO surface. This initiates an autocatalytic degradation cycle involving interface breakdown, Co dissolution, and harmful HF generation, which further accelerates electrolyte consumption through parasitic reactions. To interrupt this destructive cycle while preserving commercial feasibility, researchers have turned to additive engineering as a practical and economical approach. Specially designed electrolyte additives can effectively disrupt the chain of degradation processes under high‐voltage conditions [149, 150]. For instance, Si‐containing compounds have demonstrated effectiveness in suppressing HF formation, though their detailed protection mechanisms require further clarification [151]. Additionally, anhydride‐based additives have been used to remove trace water from the electrolyte, which otherwise promotes LiPF_6_ hydrolysis and subsequent HF production [152].

**FIGURE 15:**
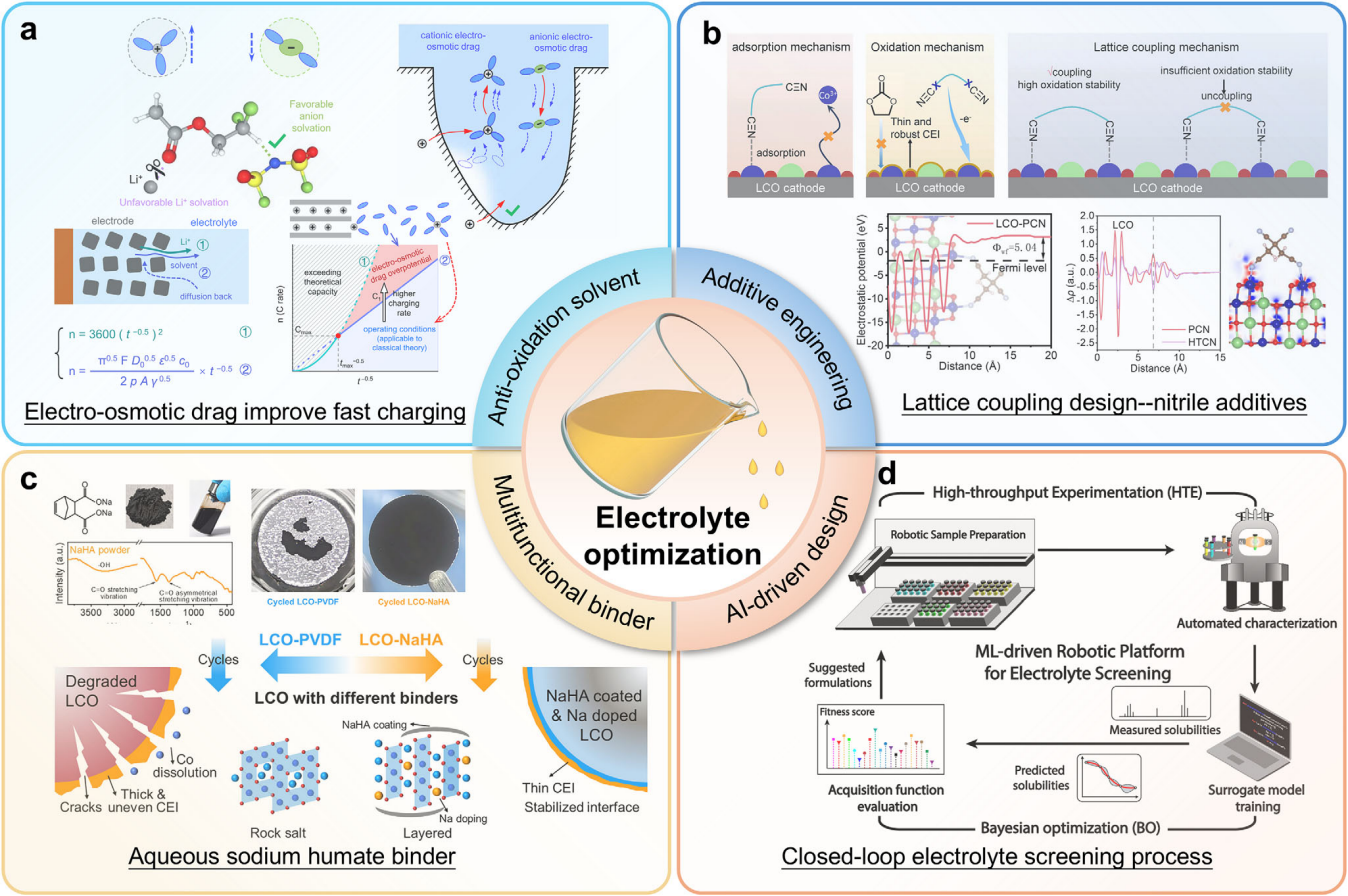
Advantages of electrolyte optimization. (a) Anti‐oxidation solvent. Reproduced with permission [153]. Copyright 2025, The American Association for the Advancement of Science. (b) Additive engineering. Reproduced with permission [154]. Copyright 2025, Cell press. (c) Multifunctional binder. Reproduced with permission [155]. Copyright 2025, WILEY‐VCH. (d) Reproduced under terms of the CC‐BY Creative Commons Attribution 4.0 International License [156]. Copyright 2024, Juran Noh, published by Springer Nature.

Electrolyte engineering plays an essential role in constructing a stable CEI with improved mechanical strength and thermal stability [157, 158, 159, 160, 161]. Figure 15 outlines key approaches to enhancing electrolyte stability in high‐voltage LIBs. (1) Anti‐oxidation solvents (Figure 15a): Fast charging in thick electrodes is often limited by electro‐osmotic drag polarization. Recent work introduces a difluorinated solvent that weakens Li‐cation solvation while strengthening anion solvation through hydrogen bonding. This design reduces polarization, enabling thick electrodes to reach 80% state of charge in 13 min. (2) Additive engineering (Figure 15b): Systematic study of additive effects on LCO has revealed a lattice‐coupling mechanism that quantifies performance enhancement. Guided by this principle, 1,2,2,3‐propanetetracarbonitrile (PCN) was identified as an effective additive, enabling stable cycling of 4.55 V LCO pouch cells at both 25°C and 45°C. (3) Multifunctional binder (Figure 15c): Sodium humate (NaHA), a low‐cost water‐soluble binder, provides dual modification for high‐voltage LCO. Its functional groups form strong hydrogen bonds with LCO surfaces and enable in situ sodium doping into the bulk lattice during cycling. This approach delivers exceptional cycling stability (95.1% capacity retention after 1700 cycles at 4.45 V), while also simplifying electrode recycling. (4) Artificial intelligence (AI)‐driven electrolyte design (Figure 15d): Combining high‐throughput experimentation with active learning algorithms accelerates the discovery of high‐solubility redox‐active molecules. This integrated platform has identified solvents achieving >6.20 M solubility for 2,1,3‐benzothiadiazole after testing fewer than 10% of candidate materials, demonstrating an efficient paradigm for functional electrolyte discovery. These strategies provide a comprehensive framework for developing stable electrolytes capable of supporting next‐generation high‐voltage battery systems.

**FIGURE 16:**
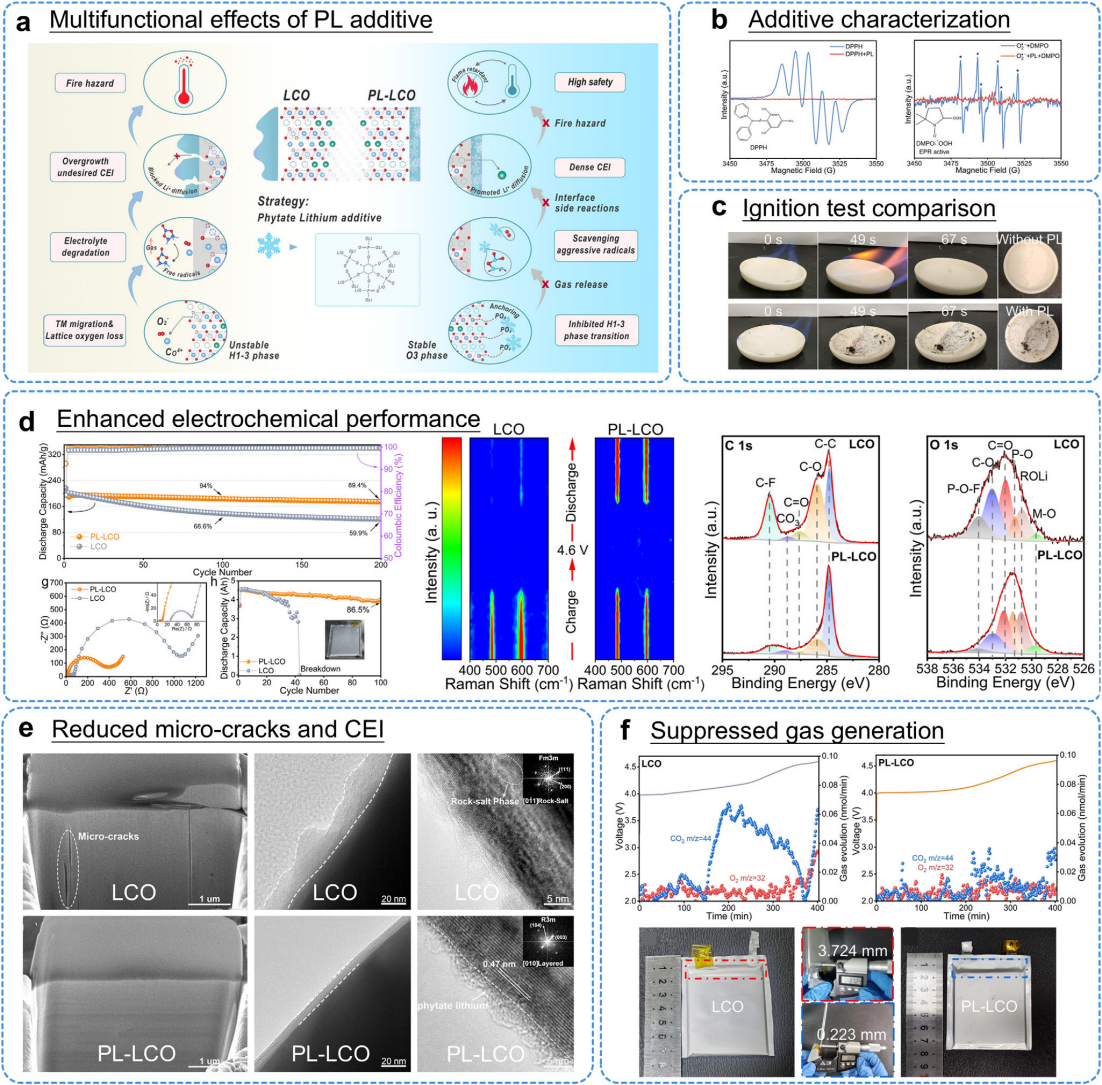
(a) Diagram depicting the multifunctional effects of PL. (b) Electron paramagnetic resonance results indicate the annihilation capability of PL. (c) Ignition test comparison of the separators wetted. (d) Electrochemical characterization, in situ Raman and X‐ray photoelectron spectroscopy (XPS). (e) Surface morphology after 50 cycles. (f) Differential electrochemical mass spectrometry (DEMS) profiles during the initial charging process up to 4.6 V and comparison of gas production in pouch cells. Reproduced with permission [162]. Copyright 2023, Royal Society of Chemistry.

As shown in Figure 16, a novel and practical approach has been developed to stabilize LCO cathodes at high voltages up to 4.6 V using phytate lithium (PL) as a multifunctional electrolyte additive [162]. The PL additive delivers multiple protective functions: (1) It forms a durable artificial CEI through strong coordination bonding between its phosphate groups and TM ions, reducing harmful phase transitions and Co dissolution under high‐voltage conditions. (2) PL effectively neutralizes oxygen radicals released from the LCO lattice at high states of charge, thereby slowing electrolyte breakdown and interface deterioration. (3) The inherent flame‐retardant properties of PL improve the thermal stability and safety of the battery system. The application of PL as a modifying agent for LCO cathodes has produced outstanding cycling performance, with nearly 90% capacity retention after 200 cycles. Furthermore, high‐energy‐density pouch cells incorporating PL‐modified LCO as the cathode demonstrate impressive capacity retention of 86.5% after 100 cycles, significantly exceeding the performance of unmodified LCO. In situ Raman spectroscopy revealed that PL‐modified LCO exhibits a lower concentration of the detrimental H1‐3 metastable phase compared to pristine LCO, confirming PL's effectiveness in suppressing detrimental phase transitions at high voltage. Additional gas evolution evaluation tests demonstrated the improved stability of PL‐modified LCO pouch cells under elevated temperature (45°C) and high‐voltage (4.65 V vs Li/Li^+^) conditions, maintaining 76% capacity after 100 cycles—substantially better than the 45% retention shown by unmodified LCO. In summary, the straightforward incorporation of PL as a versatile additive in the LCO electrode formulation effectively stabilizes the cathode structure at voltages up to 4.6 V, providing a promising strategy for practical implementation of high‐voltage LCO and other advanced cathode materials

Overall, the advancement of effective electrolyte additives represents an essential requirement for realizing high‐voltage LCO batteries with enhanced performance and safety. The capacity of these additives to stabilize CEIs and counteract the damaging effects of high‐voltage operation establishes them as indispensable components in the development of high‐energy‐density LIBs.

## 5 LCO‐Based Li‐Ion Full Cells

The commercialization of high‐energy‐density batteries necessitates a paradigm shift from material‐level optimization to scalable manufacturing solutions [163]. While academic research has made significant strides in electrode active material development, critical gaps remain in holistic cell engineering, particularly in cost‐effective design integration. This imbalance between fundamental research and practical implementation creates a bottleneck for translating laboratory breakthroughs into commercially viable energy storage systems [164, 165, 166]. The performance of Li‐ion full cells depends not only on the positive material but also on the entire battery system, including the negative, separator, electrolyte, and other components. In laboratory assessments, Li metal is commonly utilized as the counter electrode to ensure an abundant Li source, yet this setup may not faithfully mirror the performance of a complete cell with a restricted anode capacity. Hence, when assembling LCO‐based full cells, several factors merit attention: (1) Designing an appreciate electrode structure of final cell configuration; (2) Aligning the capacities/characteristics of the negative/positive (N/P); (3) Calendering electrodes for desired densities; (4) Implementing prelithiation to supply additional active Li; (5) Diagnosing and monitoring high‐voltage safety. (6) Exploring convenient external field modulation strategies. To enhance the practical application of high‐voltage LCO cathode, it is essential to optimize the entire battery system.

Achieving battery lightweighting without compromising capacity is critically important. Electrode materials selection should prioritize active materials with high tap density. Battery structural design can employ particles of varied sizes to fill in the voids more efficiently, thereby further increasing volumetric energy density. In electrode fabrication, apart from applying appropriate calendering methods, thick electrode designs can also benefit from techniques such as laser subtractive manufacturing to improve electrolyte wettability. These approaches collectively contribute to more compact and lightweight battery systems.

**FIGURE 17:**
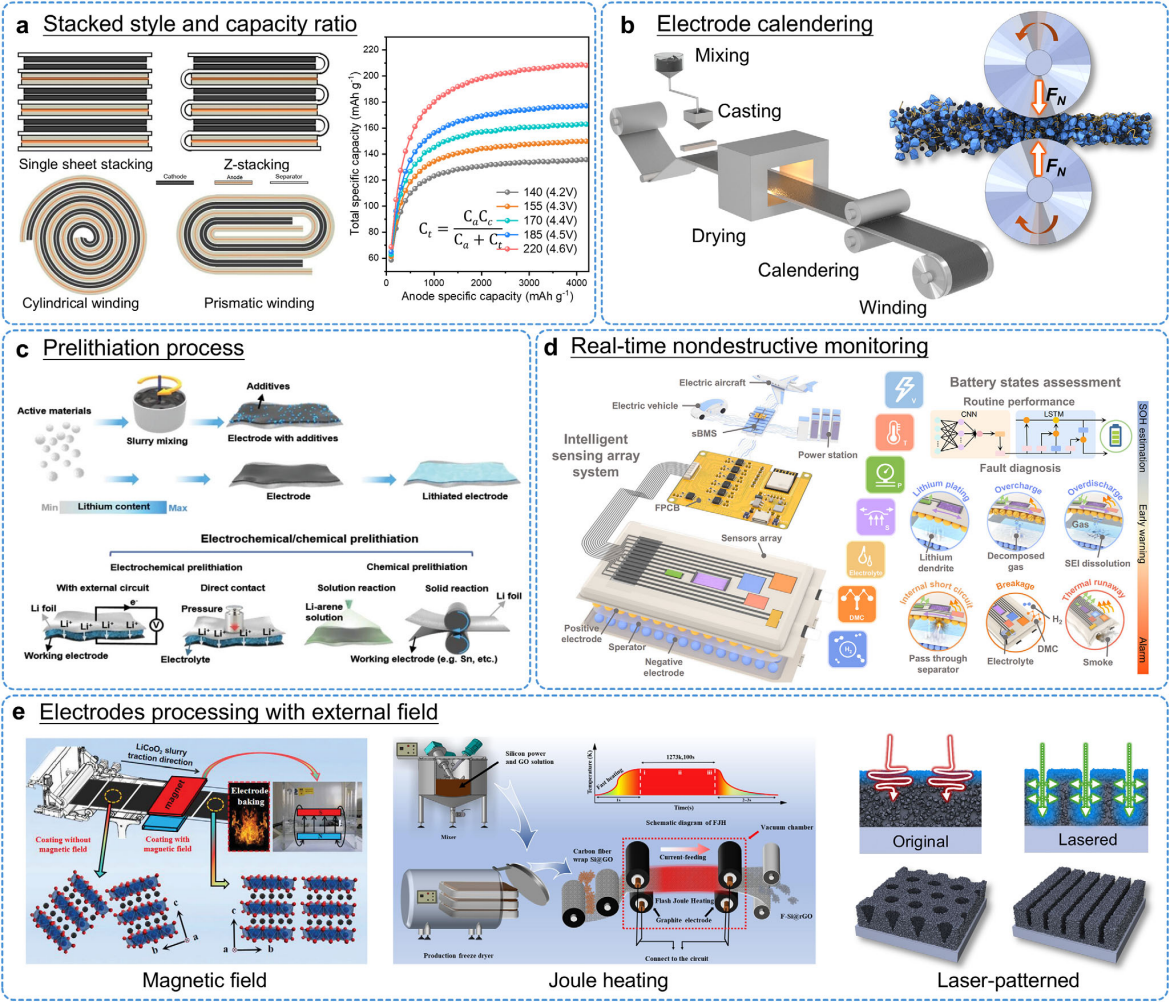
(a) Schematic showing single‐sheet‐stacked, Z‐stacked, cylindrically wound, and prismatically wound Li‐ion full cells. Reproduced under terms of the CC‐BY Creative Commons Attribution 4.0 International License [167]. Copyright 2019, Bingbin Wu, published by IOP Publishing; Total specific capacity of LCO‐based full cell as a function of the specific capacity of anode and cathode. Reproduced with permission [170]. Copyright 2018, Royal Society of Chemistry. (b) Schematic illustration of the calendering process and its effect on the electrode porosity and structure of a typical double‐sided electrode. (c) Fabrication and application of prelithiation materials, electrochemical prelithiation, and chemical prelithiation at electrode level. Reproduced with permission [171]. Copyright 2021, WILEY‐VCH. (d) Schematic illustration and actual images of the integrated intelligent sensing array system (IISAS) with multi‐feature analysis for battery state self‐assessment. Reproduced under terms of the CC‐BY Creative Commons Attribution 4.0 International License [172]. Copyright 2025, Nuo Sun, published by Springer Nature. (e) Schematic diagram of electrodes processing with external magnetic field: external magnetic field. Reproduced with permission [173]. Copyright 2023, WILEY‐VCH; flash Joule heating‐induced welding. Reproduced with permission [174]. Copyright 2021, Elsevier; and laser‐patterned manufacturing.

The battery assembly process encompasses steps from stacking or winding to the final cell configuration (Figure 17a). Pouch cells are typically assembled by stacking multiple layers of negatives, separators, and positives using either a Z‐fold or single‐sheet stacking technique [167, 168]. In single‐sheet stacked cells, sheet separators and electrodes are alternately layered, with the stacked cell's four edges left unconstrained. Conversely, in Z‐fold stacking, negatives and positives are wrapped in a Z‐shaped manner, a process that involves numerous movements by the stacker unit, leading to a relatively slow assembly process. The jelly roll resulting from cylindrical and prismatic winding processes often harbors internal stresses due to winding tension, tabs, center pins, and winding edges. These stresses can potentially cause cell deformation during repeated cycling [169].

In a full cell configuration, the selection of suitable electrode materials and the careful tuning of N/P capacity ratio and matching their kinetics are pivotal for optimizing the overall performance of Li‐ion full cells [175, 176, 177, 178]. LCO‐based LIBs commonly integrate non‐Li metal anode materials like graphite, Li_4_Ti_5_O_12_ (LTO) and Si/C composite. As shown in Figure 17a, in full cells based on LCO, the overall specific capacity (*C*_t_) primarily relies on the specific capacities of the positive (*C*_p_) and negative (*C*_n_) [170]. Capacities must align to ensure maximum utilization of both electrodes, achievable by adjusting the mass ratio based on their specific capacities [179]. Proper matching of the specific capacities of the positive and negative gives a clear enhancement in the total specific capacity. While Si/C composite anodes can achieve high specific capacities (around 800–1200 mAh g^−1^), the full‐cell capacity is ultimately constrained by the fixed capacity of the LCO cathode, creating a performance bottleneck [180]. Therefore, a balanced design integrating high‐voltage LCO cathodes with advanced high‐capacity anodes presents a promising strategy for developing next‐generation LIBs with superior energy density. To optimize full cell performance, careful matching of charge transfer kinetics and ionic diffusion rates between both electrodes is essential, as mismatched transport properties can limit the realization of theoretically achievable energy densities.

LIBs are widely used in consumer electronics like laptops and smartphones, where the volumetric energy density is extremely important. While the large single‐crystal size of LCO contributes to high tap density, a bimodal particle size distribution remains crucial to further bolster volumetric energy density. Electrode calendering, a crucial production step impacting porosity, adhesion, thickness, wettability, charge transfer properties, and coating uniformity, plays a significant role (Figure 17b) [181]. Optimal calendering can balance volumetric energy density, cycling stability, rate capability, and bonding strength between active materials, conductive carbons, binders, and the current collector [182, 183, 184]. Furthermore, single‐crystal cathode materials allow for higher electrode compaction density and are less prone to cracking when rolled into electrodes.

During the initial charging process, partial Li^+^ ions are inevitably consumed and generate a SEI layer on the surface of negative. This formation results in the loss of active Li^+^ ions, leading to a significant decrease in the capacity and energy density of full cells [185]. To address this issue, prelithiation, a method that introduces additional active Li^+^ ions to the battery, stands out as a highly promising strategy (Figure 17c) [171]. The prelithiation process necessitates careful consideration of factors such as available Li^+^ ion capacity, prelithiation efficiency, residues, and side reactions. To develop prelithiation additives with substantial Li^+^ ion capacities, metallic Li is commonly employed as the primary material. Effective prelithiation can notably improve Coulombic efficiency, reversible capacity, and cycling stability of the full cell [186, 187]. The advancement of prelithiation technology is paramount and pressing for the realization of the next generation of high‐energy‐density Li‐ion full cells.

Smart battery management systems epitomize extreme optimization through the integration and real‐time analysis of ultra‐heterogeneous data streams (including ultrasonic, thermal, voltage, and current data) to achieve sophisticated health state prediction, operational optimization, and early accident warning, thereby ensuring ultimate safety and reliability [188, 189, 190, 191, 192, 193, 194, 195]. Moving beyond existing monitoring technologies such as fiber optic or acoustic methods [196, 197, 198, 199, 200], which often suffer from complex equipment and challenges in long‐term deployment, a breakthrough solution has emerged: an ultra‐thin, flexible, and multifunctional sensor array that can be directly embedded into a LIB's packaging film (Figure 17d) [172]. This innovative array is capable of synchronously monitoring a comprehensive suite of parameters including temperature, pressure, strain, hydrogen gas, dimethyl carbonate vapor, and liquid electrolyte presence. Fabricated by CO_2_ laser etching of an aluminum‐polymer composite foil to create interconnect lines, and embedded within PET layers with a polyurethane film encapsulation, the entire system adds a negligible weight of only 0.049 g and occupies a mere 16.844 mm^3^, resulting in a minimal impact on the battery's energy density and electrochemical performance, while its anti‐interference stability surpasses that of commercial discrete sensors and is compatible with various battery structures.

Furthermore, there is an urgent need to explore convenient zero‐cost or near‐zero‐cost modification strategies, which are crucial for industrial development of high‐energy electrode materials technology. External field modulation strategies (magnetic fields, Joule heating, etc.) represent a non‐contact energy transfer method with extensive prospects in micro‐nano‐device fabrication [201, 202]. By altering the physicochemical properties of electrode materials through the application of external fields during the electrode preparation process, these strategies contribute to various aspects such as controlled material preparation, enhanced cycling stability, and safety assurance.

The strategic application of magnetic fields has emerged as a promising approach to enhancing Li‐ion full‐cell performance through multiple physical mechanisms, including magnetic force, magnetization, magnetohydrodynamic effects, and spin polarization [203, 204]. As shown in Figure 17e, Zhang et al. demonstrated this concept by aligning LCO crystal phases using a moderate 500 mT magnetic field, which significantly improved the electrochemical behavior of LCO||graphite pouch cells. Magnetic fields increase the rate of the Li^+^ deintercalation processes, optimize the diffusion path, improve the discharge capacity, and reduce electrode polarization. This approach not only boosts rate capability but also enhances thermal safety, offering a non‐invasive method to upgrade conventional battery systems [173]. Additionally, the magnetic field alignment induces the formation of vertically oriented channels within the electrode architecture, creating optimized ionic diffusion pathways. This unique magnetically templated microstructure accelerated charge transfer kinetics through aligned conduction pathways, reaching three times higher areal capacity (>12 mAh cm^−2^ vs traditional <4 mAh cm^−2^) [205].

Flash Joule heating treatment is considered another effective method [206, 207, 208]. As shown in Figure 17e, Yang et al. controlled the thermal interaction between carbon and silicon phases using ultra‐fast Joule heating, reinforcing the structural stability of graphene matrices [174]. This approach effectively mitigates phase segregation issues caused by varying wettability during traditional heat treatment processes, ensuring strong bonding between graphene and silicon. For spent graphite electrodes, Dong et al. efficiently restored their performance using flash Joule heating [209]. The graphite electrodes are rapidly regenerated within 0.1 s with no pollutant emissions. Joule heating technology enables defect repair and crystal structure reconfiguration in graphite, providing substantial current for super‐fast annealing processes and eliminating SEI coating formation.

The overall performance of high‐energy‐density electrodes depends significantly on their structural design across both micro‐ and macro‐scales. Key factors include electrode porosity, tortuosity, and the integrity of the conductive network. Advanced manufacturing techniques such as ultra‐high‐pressure calendering, laser ablation, and gradient electrode stacking allow precise control over ion and electron transport pathways [210]. These methods help realize the theoretical performance of advanced cathode materials. For example, femtosecond laser ablation has been used to create well‐defined micro‐grooves on thick cathodes (∼280 µm). These grooves serve as integrated electrolyte reservoirs [211]. More importantly, Li^+^ ions can directly enter the electrode through the groove sidewalls. This significantly reduces transport distances and lowers electrode tortuosity. In a high‐loading electrode (60 mg cm^−2^), this strategy reduced tortuosity by 20.2%, boosted power density by 100%–200%, and increased the specific capacity at 1.5C from 8.48 to 84.86 mAh g^−1^, which is an almost tenfold improvement. These enhancements were achieved without compromising electronic conductivity or mechanical stability. Imaging of lithium concentration distribution further confirmed that the grooved structure promotes uniform reaction throughout the electrode, which is especially important under high‐current operation.

## 6 Conclusion and Perspectives

This review provides a systematic examination of the operational principles, degradation mechanisms, and performance enhancement strategies for high‐voltage LCO cathodes in LIBs. Advancing the energy density of LIBs necessitates overcoming challenges associated with elevated operating voltages. A thorough understanding of cathode working mechanisms, failure pathways, and established modification approaches is therefore fundamental to progress in energy storage technology. The development of effective strategies to improve cathode materials under high‐voltage conditions plays a vital role in the evolution of advanced battery systems. Through careful optimization of bulk lattice properties and surface engineering, it becomes possible to enhance the specific capacity, operational reliability, and cycle life of high‐voltage cathode materials. These advancements hold significant value for the development of lightweight portable electronics and next‐generation energy storage solutions, thereby supporting the broader adoption of sustainable energy and accelerating the transition to clean energy systems.

**FIGURE 18:**
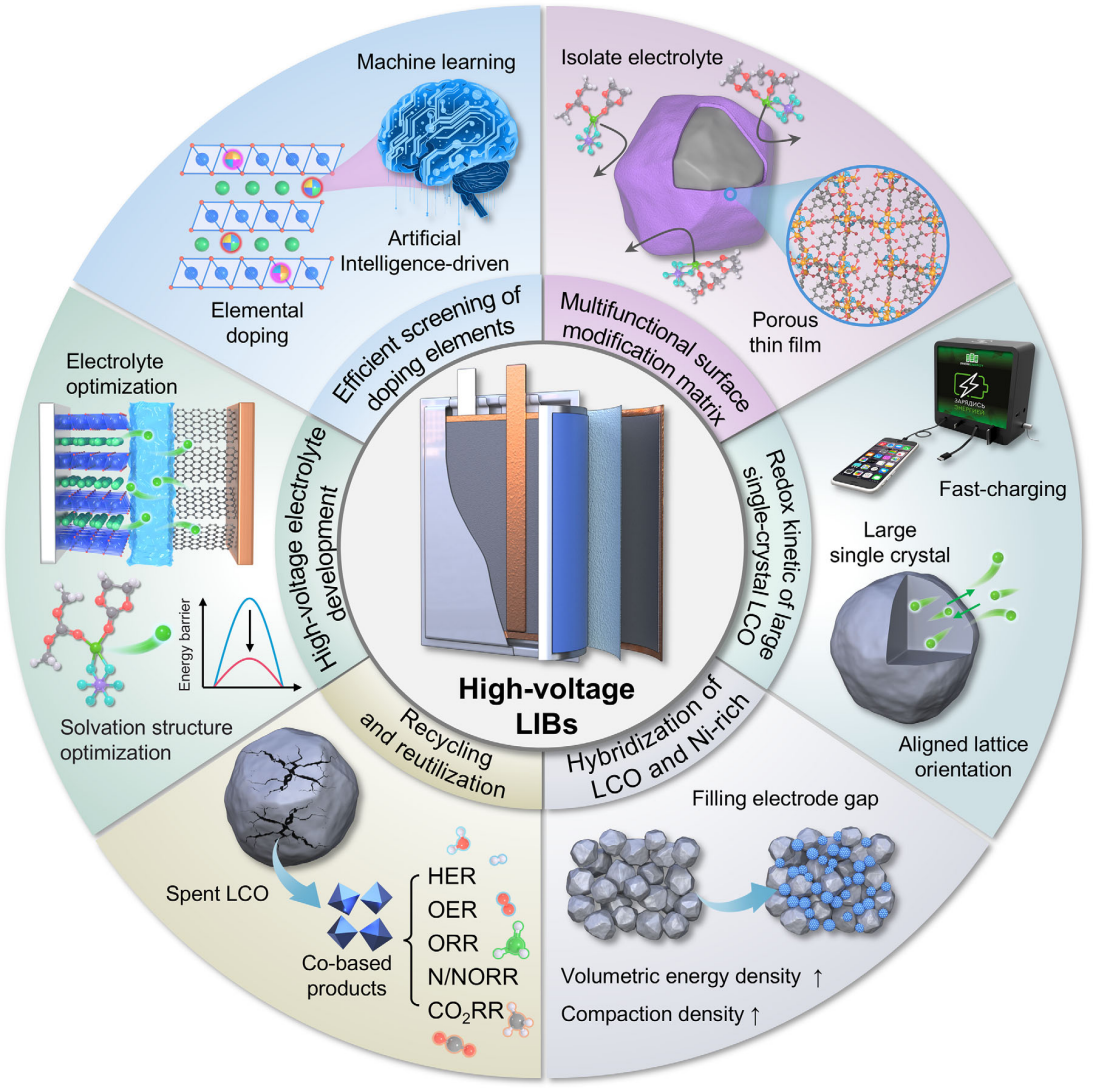
The remaining challenges and potential solutions of high‐voltage LCO‐based LIB systems.

Among various layered transition metal oxide cathodes, LCO distinguishes itself through well‐defined voltage plateaus, high volumetric density, superior energy density, and straightforward synthesis. These characteristics maintain its position as the leading commercial cathode material today. Nevertheless, enabling stable LCO performance at or above 4.6 V requires continued scientific investigation and technological development. This review accordingly concentrates specifically on failure mechanisms and modification methodologies for LCO cathodes in high‐voltage LIB configurations. It aims not only to deepen the structural understanding of LCO under extreme conditions but also to guide the rational design of stable high‐voltage LCO variants in the future. Although substantial progress has been made in elucidating failure mechanisms and improving the performance of high‐voltage LCO in recent research, several unresolved challenges persist in LCO‐based battery systems, as summarized in Figure 18. In the following section, we discuss remaining challenges and potential solutions for developing high‐voltage LCO cathodes suitable for next‐generation LIBs.

1. Advanced screening of dopants and multi‐element combinations for high‐voltage LCO. Foreign‐ion doping has demonstrated considerable potential for stabilizing LCO under high‐voltage conditions. However, the selection of dopant species, their concentrations, and synergistic combinations currently rely heavily on experimental trial and error, which is both time‐intensive and inefficient. Given the largely empirical nature of dopant selection for high‐voltage LCO cathodes, there is a pressing need for more efficient and systematic screening methods. Integrating high‐throughput DFT calculations with machine learning offers a promising path forward. Such an approach can help build extensive databases linking structural characteristics to dopant properties, enabling the identification of optimal doping schemes through appropriate descriptors [212]. This methodology also allows rapid exploration of a wide range of multi‐element combinations, helping prioritize promising higher‐order compositions and predict key material properties. Overcoming current limitations (such as insufficient diverse datasets and limited model interpretability) will be essential to advancing machine learning‐assisted design of doped LCO cathodes.
2. Design of multifunctional surface coating matrices. Surface coatings can effectively minimize direct contact between the cathode and electrolyte, stabilize lattice oxygen, suppress TM dissolution, and inhibit oxidative decomposition—collectively enabling stable high‐voltage operation of LCO. Current coating materials mainly include oxides, phosphates, fluorides, and carbon‐based substances, which are typically amorphous and structurally mismatched with the layered framework of LCO. The nanocrystalline nature and abundant grain boundaries in these conventional coatings tend to extend Li^+^ ion transport pathways and limit Li^+^ kinetics. Emerging materials such as metal‐organic frameworks (MOFs), covalent organic frameworks (COFs), and zeolites (featuring well‐defined pore structures, high crystallinity, and good structural compatibility) present an attractive alternative as multifunctional coating matrices for high‐voltage cathodes. These proposed coating candidates are expected to simultaneously offer physical isolation, enhance interfacial charge transfer, maintain efficient Li^+^ transport channels, suppress transition metal dissolution, and accommodate surface‐unstable lattice oxygen.
3. Necessity of high‐voltage electrolyte development. Under high‐voltage conditions, parasitic reactions between highly reactive Co^4+^ species and organic electrolytes result in oxidative decomposition of both the electrolyte and the LCO surface. Furthermore, dissolved Co^2+^ ions enter the electrolyte solvation structure (forming complexes such as Co^2+^(EC)_n_ or Co^2+^(DMC)_n_, etc.) which can further accelerate electrolyte oxidation. These challenges highlight the urgent need for advanced high‐voltage electrolyte systems that can suppress such detrimental processes and maintain electrode integrity. Future research should focus on designing durable electrolyte formulations based on a fundamental understanding of electrolyte consumption mechanisms. This includes identifying solvents with high oxidation stability and broad electrochemical windows to replace conventional carbonate‐based solvents. Promising candidates such as sulfones, fluorinated solvents, and nitrile‐based compounds offer significant potential to improve high‐voltage battery performance and extend cycle life.
4. Enhancing redox kinetics in large single‐crystal LCO. Current research efforts predominantly aim to improve cycling stability and volumetric energy density in high‐voltage LCO. The development of single‐crystal LCO with particle sizes exceeding 20 µm, combined with surface coatings and elemental doping, has shown encouraging results in addressing these objectives. However, the increased particle size significantly lengthens Li^+^ ion diffusion pathways, thereby limiting reaction kinetics and high‐rate performance. Nonetheless, the growing demand for fast charging rates above 2C (corresponding to charge times less than 30 min) in portable electronic devices necessitates improved redox kinetics in large single‐crystal LCO. Potential strategies to overcome these limitations include optimizing foreign‐ion doping to expand interlayer spacing and lower Li^+^ diffusion barriers, reconstructing the cathode surface to form spinel‐like phases with 3D Li^+^ transport channels, and applying porous surface coatings that promote rapid Li^+^ ion desolvation
5. Sustainable LCO recycling and reutilization. Co, a crucial component of LIBs, is both expensive and subject to supply chain risks due to geopolitical issues and limited abundance. Efficient recycling and reutilization of Co can address these challenges. The failure of LCO primarily involves Li loss, structural degradation, and the accumulation of surface CEI. Re‐lithiation, achieved through electrodeposition and low‐temperature binary Li‐containing molten salt systems, is an effective method for regenerating the spent LCO cathodes and recovering the original layered structure behavior [213, 214, 215, 216, 217, 218, 219, 220]. Additionally, a low‐temperature thermochemistry route, involving thermal reduction, can be explored for the selective recycling of Co metals from spent LCO [221]. The Co‐based products obtained through this process, including spinel Co_3_O_4_, CoO, and related compounds, can serve as highly active electrocatalysts for various energy conversion reactions, such as HER, OER, ORR, N/NORR, and CO_2_RR [222, 223]. These approaches contribute to creating an innovative approach within the circular economy, transforming waste into a valuable electrochemical production and conversion system.
6. Hybridization of large‐size LCO and small‐size Ni‐rich single crystals. Hybridizing large single crystal LCO with small Ni‐rich single crystals offers a promising strategy to improve electrode compaction density and enhance the volumetric energy density of LIBs. By filling the gaps within the large LCO crystals with small Ni‐rich counterparts, this approach not only increases electrode compaction density and energy density but also reduces the cost of LIBs by utilizing lower‐cost Ni resources and Ni‐rich cathode materials. However, achieving an optimal balance in particle sizes and the mixing ratio of large and small crystals is crucial. This balance is necessary to simultaneously enhance compaction density and performance, but it poses a significant challenge. Future research should focus on addressing this issue to realize the full potential of this hybrid approach.

## Author Contributions

L.Z. contributed to conceptualization, formal analysis, figure and writing of the original draft. Y.R., L.H., R.H., and L.T. contributed to literature review and data collection. H.P. contributed to conceptualization, supervision, review, and editing. H.H. contributed to conceptualization, supervision, writing, review, and editing.

## Conflicts of Interest

There are no conflicts to declare.

## Data Availability

The data that support the findings of this study are available from the corresponding author upon reasonable request.
